# Advances in Machine Learning-Enhanced PBPK Models for Brain-Targeted Drug Delivery via Nanocarriers: A Comprehensive Review

**DOI:** 10.3390/jfb17080377

**Published:** 2026-08-03

**Authors:** Hanwen Hu, Ya Wang

**Affiliations:** 1 J. Mike Walker ‘66 Department of Mechanical Engineering, Texas A&M University, College Station, TX 77843, USA; hanwen@tamu.edu; 2 Department of Electrical and Computer Engineering, Texas A&M University, College Station, TX 77843, USA; 3 Department of Biomedical Engineering, Texas A&M University, College Station, TX 77843, USA; 4 Department of Computer Science and Engineering, Texas A&M University, College Station, TX 77843, USA

**Keywords:** nanostructured drug-delivery systems, drug-loaded nanocarriers, brain-targeted delivery, blood–brain barrier, physiologically based pharmacokinetic (PBPK) modeling, machine learning, ML–PBPK hybrid models, pharmaceutical nanomedicine

## Abstract

Nanostructured drug-delivery materials—liposomes, polymeric nanoparticles, dendrimers, and inorganic carriers—have become central to pharmaceutical strategies for crossing the blood–brain barrier (BBB), where most candidate therapeutics fail to reach their targets. Their biological performance hinges on a coupled chain of vascular transport, BBB translocation, tissue diffusion, cellular uptake, and intracellular release, each of which is shaped by the nanocarrier’s size, surface chemistry, charge, and ligand functionalization. Physiologically based pharmacokinetic (PBPK) models describe this chain mechanistically but are limited by parameter uncertainty, simplified representations of the BBB, and coarse regional resolution. Machine learning (ML) can close these gaps by extracting nonlinear structure–transport–exposure relationships from heterogeneous experimental and clinical datasets. This review examines emerging ML–PBPK hybrid frameworks for predicting the brain biodistribution of nanostructured drug carriers. We compare regression, kernel, and deep learning approaches for parameter inference, model correction, and surrogate modeling; assess strategies for feature selection, uncertainty quantification, and interpretability; and discuss documented failure cases that bound the conditions under which these methods can be trusted. The review closes with recommendations on dataset standardization, software platform selection, and the responsible use of generative AI in pharmaceutical modeling, thus providing guidance for translating nanostructured material design into safer, more effective brain-targeted therapies.

## 1. Introduction

**Figure 1:**
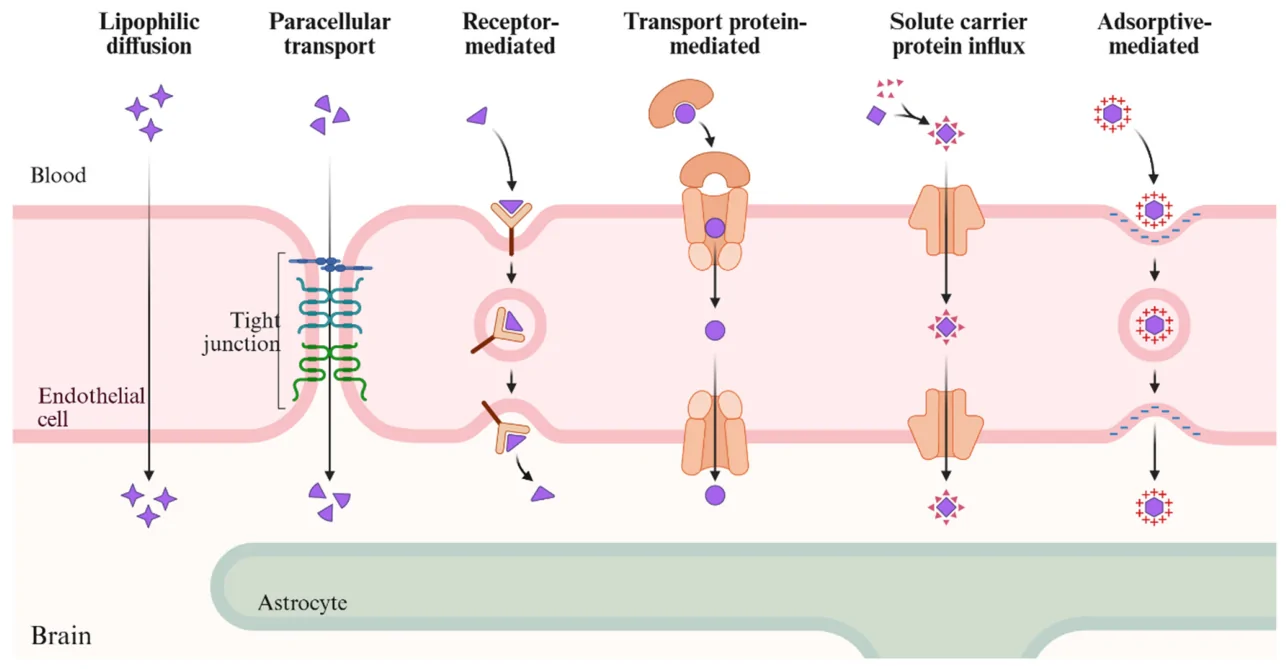
Representative DNC transport mechanisms across the BBB, including passive diffusion, carrier-mediated transport, adsorptive- and receptor-mediated transcytosis, efflux by endogenous transporters (e.g., P-gp, BCRP), and exogenously stimulated permeabilization (e.g., focused ultrasound, magnetic guidance). These mechanisms define the parameter space (PS-type terms, efflux clearances, and ligand–receptor kinetics) that ML–PBPK frameworks attempt to infer or surrogate; their relative contribution depends on DNC size, surface chemistry, and local BBB state.

Delivering therapeutics to the brain remains a significant challenge due to the restrictive blood–brain barrier (BBB), which blocks over 98% of small molecules and allows only essential nutrients through [1]. Drug-loaded nanocarriers (DNCs) offer a promising strategy to overcome this limitation by exploiting multiple transport routes across the BBB, including paracellular passage, passive diffusion, and active transcytosis (Figure 1). Surface functionalization with ligands (e.g., peptides, antibodies) or charge-based interactions can further enhance selective uptake. At the same time, external stimuli such as ultrasound or magnetic fields may transiently increase permeability [2].

In addition to improving BBB penetration, DNCs can protect therapeutics from degradation, prolong systemic circulation, bypass efflux pumps, and enable sustained or stimuli-responsive release. Functionalized DNCs also support theranostic applications by integrating drug delivery with real-time imaging [3]. Together, these advances underscore the pivotal role of DNCs in facilitating safe, efficient, and targeted brain drug delivery.

### 1.1. Challenges in Modeling Brain-Targeted Delivery Efficiency

**Figure 2:**
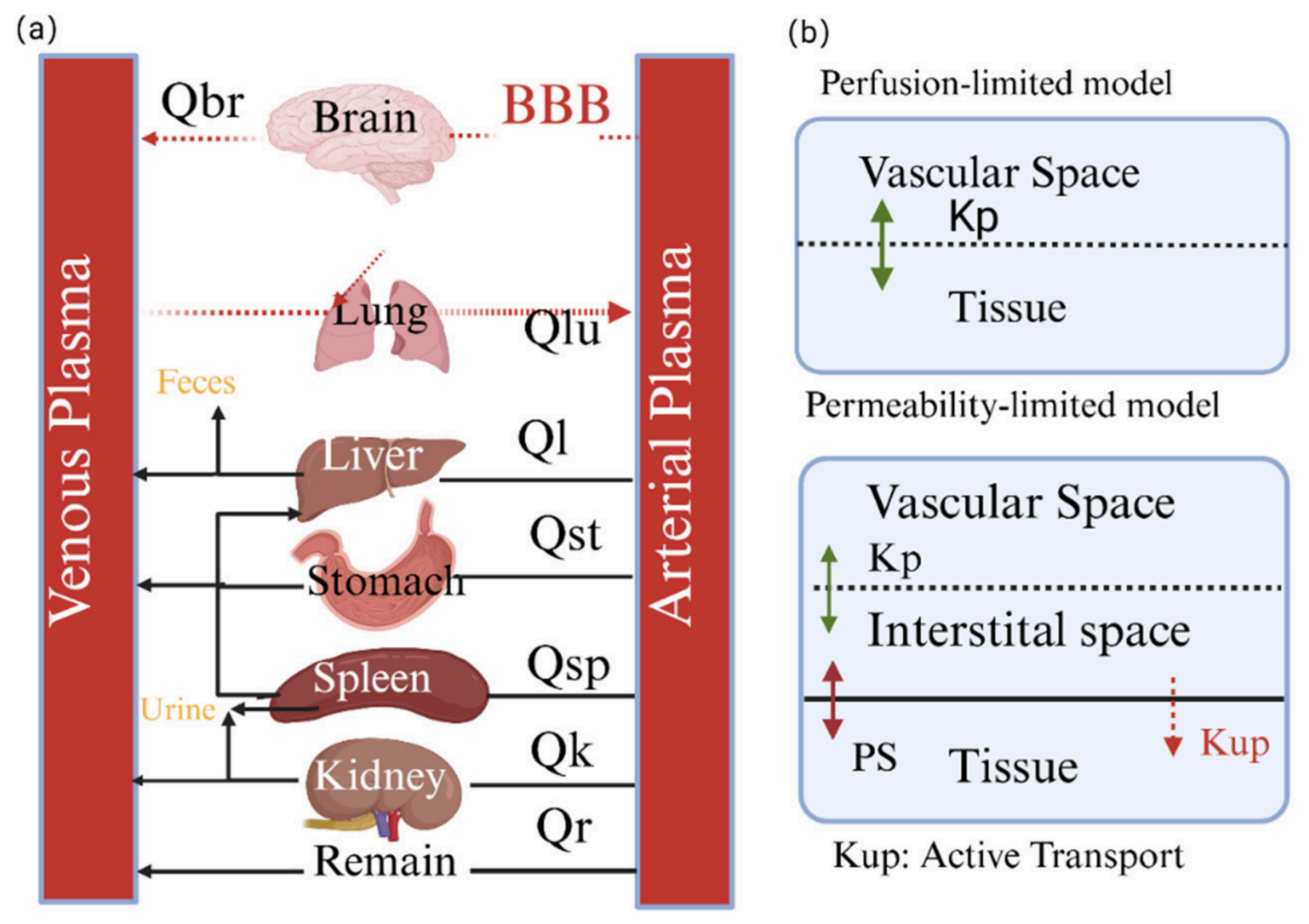
(**a**) Whole-body PBPK model contains organ and plasma components, where Q denotes blood flow rates to each organ. (**b**) Comparison of two tissue distribution models: the perfusion-limited model, where drug/DNC transport is primarily governed by blood flow and partition coefficient Kp, and the permeability-limited model, where transport is further constrained by permeability-surface (PS), interstitial barriers, and potential active uptake processes Kup.

Accurately predicting the absorption, distribution, metabolism, and excretion (ADME) of DNCs is crucial for optimizing brain delivery, informing dosing strategies, and minimizing systemic toxicity. Modeling approaches span molecular dynamics simulations, which capture atomic-level interactions at the BBB, to multiscale frameworks that link molecular transport with tissue-level distribution [4]. Among these, physiologically based pharmacokinetic (PBPK) modeling has emerged as a powerful tool because it mechanistically represents biological processes (Figure 2), incorporates DNC-specific properties, and accounts for inter-individual variability [5]. Hybrid PBPK models, which integrate both perfusion- and permeability-limited kinetics, are especially relevant for simulating the BBB’s restrictive and heterogeneous nature [6].

However, current PBPK models often treat the BBB as a static barrier, overlooking its dynamic regulation by tight junctions, efflux transporters, and receptor-mediated transcytosis [7]. These processes are nonlinear, context-dependent, and challenging to parameterize due to limited or incomplete experimental data [8]. Parameter estimation remains a key challenge: while in vitro assays, computational predictions, and public databases provide inputs, they rarely capture variability in brain vasculature, disease states, or transport dynamics. Even advanced 3D vascular models and consideration of administration routes cannot fully address these limitations [9]. Consequently, brain PBPK models are constrained by physiological complexity, uncertainty in parameter identification, and insufficient datasets. Machine learning (ML) offers a promising solution by integrating diverse preclinical, clinical, imaging, and omics datasets; compensating for missing data; and improving parameter estimation through advanced optimization strategies to increase predictive accuracy.

### 1.2. The Potential of ML–PBPK in Predicting Brain-Targeted Delivery Efficiency

Integrating ML with PBPK modeling offers a powerful strategy for improving predictions of DNC distribution in the brain. ML can automate parameter estimation, uncover hidden patterns from large datasets, and dynamically update model relationships, thereby bridging gaps between in vitro and in vivo data [10,11,12,13]. Deep learning methods, such as recurrent neural networks, are particularly suited to capturing nonlinear dynamics and temporal changes in brain physiology.

However, ML–PBPK modeling also faces key challenges. Black-box algorithms lack mechanistic transparency, which complicates their regulatory acceptance. Risks of overfitting and the need for large, high-quality datasets further limit their generalizability, while the structural diversity of DNCs complicates feature selection [14]. Developing robust databases that capture BBB variability across disease states and drug classes remains a pressing need.

This review examines how ML methodologies are being integrated into PBPK modeling to enhance prediction accuracy for brain-targeted DNC delivery. We systematically evaluate existing approaches, compare them with conventional PBPK frameworks, and highlight emerging opportunities and challenges for advancing ML–PBPK modeling in nanomedicine.

## 2. ML–PBPK Review

### 2.1. Existing ML–PBPK Reviews

Table 1 summarizes representative ML–PBPK-related reviews that collectively demonstrate how machine learning can enhance PK parameter prediction, support descriptor selection and QSAR modeling, assist model-informed drug development, and analyze large nano-toxicology and nanomedicine datasets. However, most of these reviews are restricted to specific therapeutic areas or systemic ADME endpoints, rely on limited or biased datasets, and seldom provide a systematic, quantitative comparison of ML–PBPK model performance across different parameter categories or brain-related PK outputs. Consequently, they offer little dedicated discussion of ML–PBPK frameworks for brain-targeted DNCs or multi-route brain distribution modeling, leaving a significant gap that our review aims to address.

Three crucial gaps emerge that are evident in Table 1; addressing these gaps distinguishes the present review. First, none of the surveyed reviews provides a quantitative cross-study comparison of ML–PBPK predictive performance for brain-specific endpoints (Kp, uu, brain, BBB PSeff, and logBB) reported alongside dataset size and validation split, which are the conditions under which AUC and R^2^ values are interpretable. Second, prior reviews discuss either ML or PBPK or nanocarrier physiochemistry in isolation, with very limited coverage of the receptor-mediated and adsorptive transcytosis pathways that dominate brain delivery for surface-functionalized nanocarriers. Third, none provides an explicit critical assessment of failure modes—cases in which ML-augmented PBPK models mis-predicted brain exposure by an order of magnitude or violated mass balance—which is precisely the information needed to bound the credible scope of the approach for nanocarrier design. The present review is structured to address these three gaps directly.

### 2.2. The Focus of This Review

We identified the primary literature through a structured search of PubMed, Web of Science, and Scopus, covering peer-reviewed work from January 2008 through December 2025. The search targeted studies that combined a machine learning method with a PBPK or compartmental brain-PK model for a CNS- or BBB-relevant application; inclusion did not require a focus on nanocarriers. We retained a study only if it specified an actual ML algorithm and reported at least one quantitative performance metric (AUC, RMSE, R^2^, or similar), connected that ML output to a PBPK or compartmental brain-PK component rather than stopping at a stand-alone QSAR, and disclosed a traceable data source or shared its code. Conference abstracts, editorials, ML studies with no PBPK linkage, and mechanistic PBPK studies that used no ML were excluded. For every study that satisfied these criteria, we recorded the validation strategy, dataset size, and handling of class imbalance; the origin of its data, whether in vitro, in vivo, clinical, or in silico; and whether it was tested against independent or prospective data. Those notes are what make an otherwise heterogeneous body of work comparable in the sections that follow.

We then grouped the retained studies in three ways. One axis is the ML algorithm family—linear and kernel methods, tree ensembles, and deep architectures spanning feed-forward, recurrent, and graph- or sequence-based networks—and for each, we tracked dataset size, the training/validation split, and any reported uncertainty estimates. A second axis is the brain-specific PK parameter a study tries to predict or constrain: the BBB permeability–surface-area product (PSeff), the unbound brain-to-plasma partition coefficient (Kp, uu, brain), logBB, or the kinetic parameters of receptor-mediated transcytosis. The last is a spatial scale, which extends from lumped whole-brain compartments to three-dimensional vascular geometries reconstructed from MRI or CT angiography. Where a study reported a negative or null result, we deliberately set it next to the positive-outcome work, since the publication bias in this field has left few documented failure modes to learn from.

Three problems recur across the studies we examined, which earlier surveys have tended to treat only in passing: the difficulty of reconciling data drawn from in vitro, in vivo, and clinical sources; the shortage of experimental datasets suitable for validating BBB transport; and the trade-off between predictive accuracy and interpretability that grows sharper as the ML models become more complex. We return to these three threads throughout this review, as they are why a systematic assessment of ML–PBPK methods for brain-targeted nanomedicine is timely.

**Figure 3:**
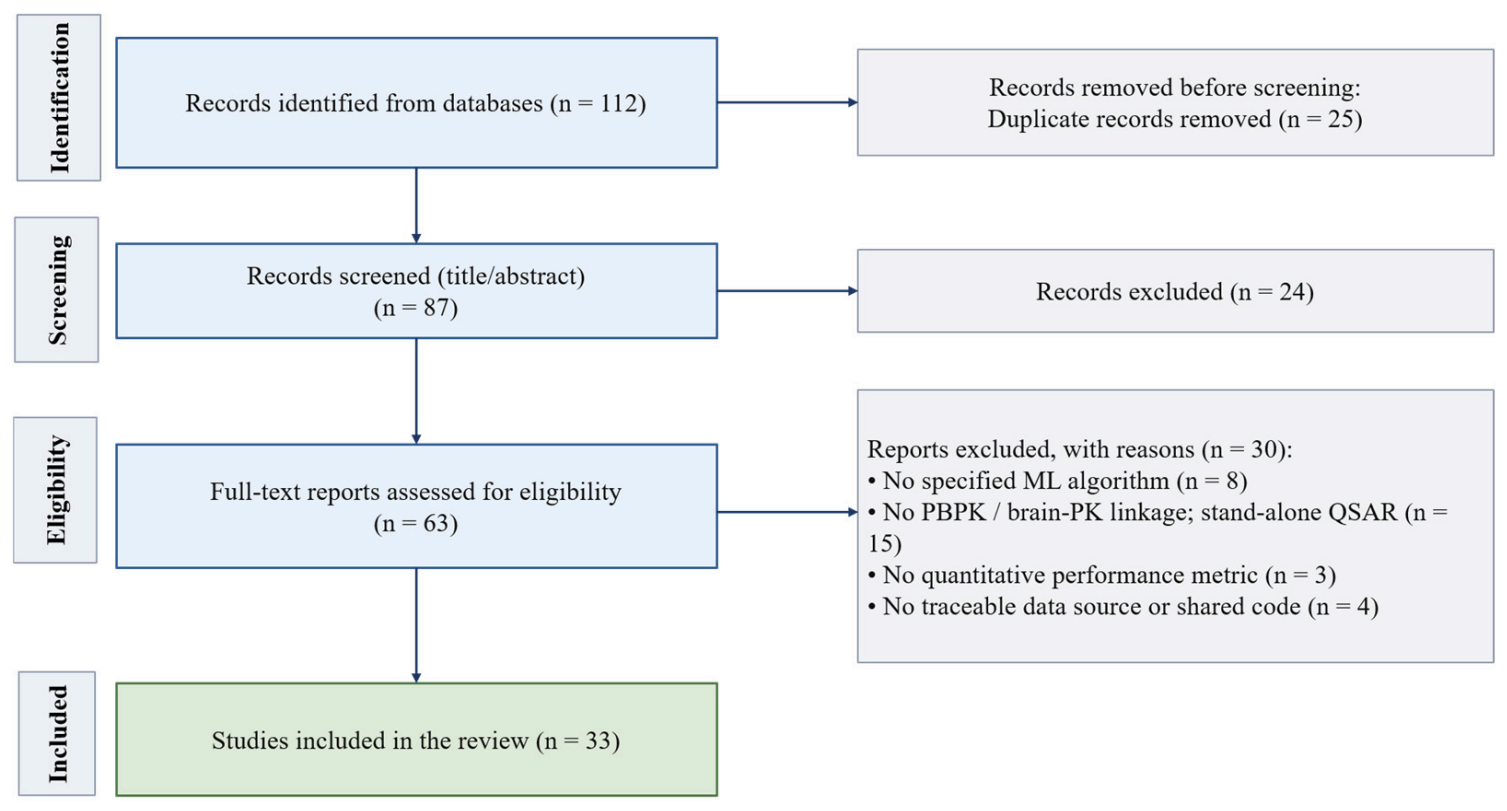
PRISMA flow diagram of the study.

Traditional whole-body PBPK models include compartments for all major organs and tissues (with dedicated brain and BBB sub-compartments), as well as blood and other fluid spaces. These models are typically governed by PK/PD sub-modules and ODE systems that use drug-specific properties (e.g., molecular weight, lipophilicity) to predict passive diffusion and distribution. We recently developed an advection–diffusion-based PBPK model to improve the prediction of brain permeability for several DNCs [23,24], providing a mechanistic framework that integrates physiological, biochemical, and drug-specific data. Active uptake processes (e.g., receptor-mediated transport) and phagocytosis modeled via an uptake rate constant (Kup) are incorporated when simulating targeted delivery mechanisms. The study selection process is summarized in Figure 3. The initial search of the three databases returned 112 records. After 25 duplicates were removed, 87 records were screened by title and abstract, of which 24 were excluded as clearly outside the scope of the review. The remaining 63 reports were assessed in full text against the inclusion criteria. Of these, 30 were excluded: 8 did not specify a machine learning algorithm, 15 had no PBPK or brain-PK linkage (including stand-alone QSAR studies), 3 reported no quantitative performance metric, and 4 did not disclose a traceable data source or shared code. This yielded 33 studies that were included in the review.

To ensure transparency and reproducibility of the literature-selection process, the inclusion and exclusion criteria applied in this review are summarized in Table 2. Studies were retrieved from PubMed, Web of Science, and Scopus, covering the peer-reviewed primary literature published between January 2008 and December 2025. To be included, a study had to combine at least one explicitly specified machine learning algorithm with a PBPK or compartmental brain-PK model for a CNS- or BBB-relevant application, report at least one quantitative performance metric (e.g., AUC, RMSE, R^2^), and disclose a traceable data source or shared code; stand-alone QSAR studies, conference abstracts, editorials, and purely mechanistic PBPK studies without an ML component were excluded. For each eligible study, we extracted the validation strategy, dataset size, class-imbalance handling, data origin (in vitro, in vivo, clinical, or in silico), and whether the model was evaluated on independent or prospective data.

## 3. Challenges of PBPK Modeling Approaches

### 3.1. PBPK Model Frameworks and CNS Physiological Barriers

Conventional PBPK models can be classified according to the assumed transport mechanism between compartments—perfusion-limited, diffusion (permeability)-limited, or active transport—with additional processes like facilitated transport, pinocytosis, and receptor-mediated transcytosis included, especially for DNCs. In a perfusion-limited model [25], rapid blood–tissue equilibrium is assumed, whereas a permeability-limited model [26] treats membrane permeation as the rate-limiting step. An active transport model [27] accounts for energy-dependent uptake processes such as receptor binding or phagocytosis [28]. The appropriate model structure often depends on DNC size: small DNCs (≤10 nm) tend to follow perfusion-limited kinetics, large particles (>100 nm) are usually diffusion-limited, and intermediate-sized DNCs (10–100 nm) may exhibit mixed behavior depending on the tissue porosity and DNC properties.

**Figure 4:**
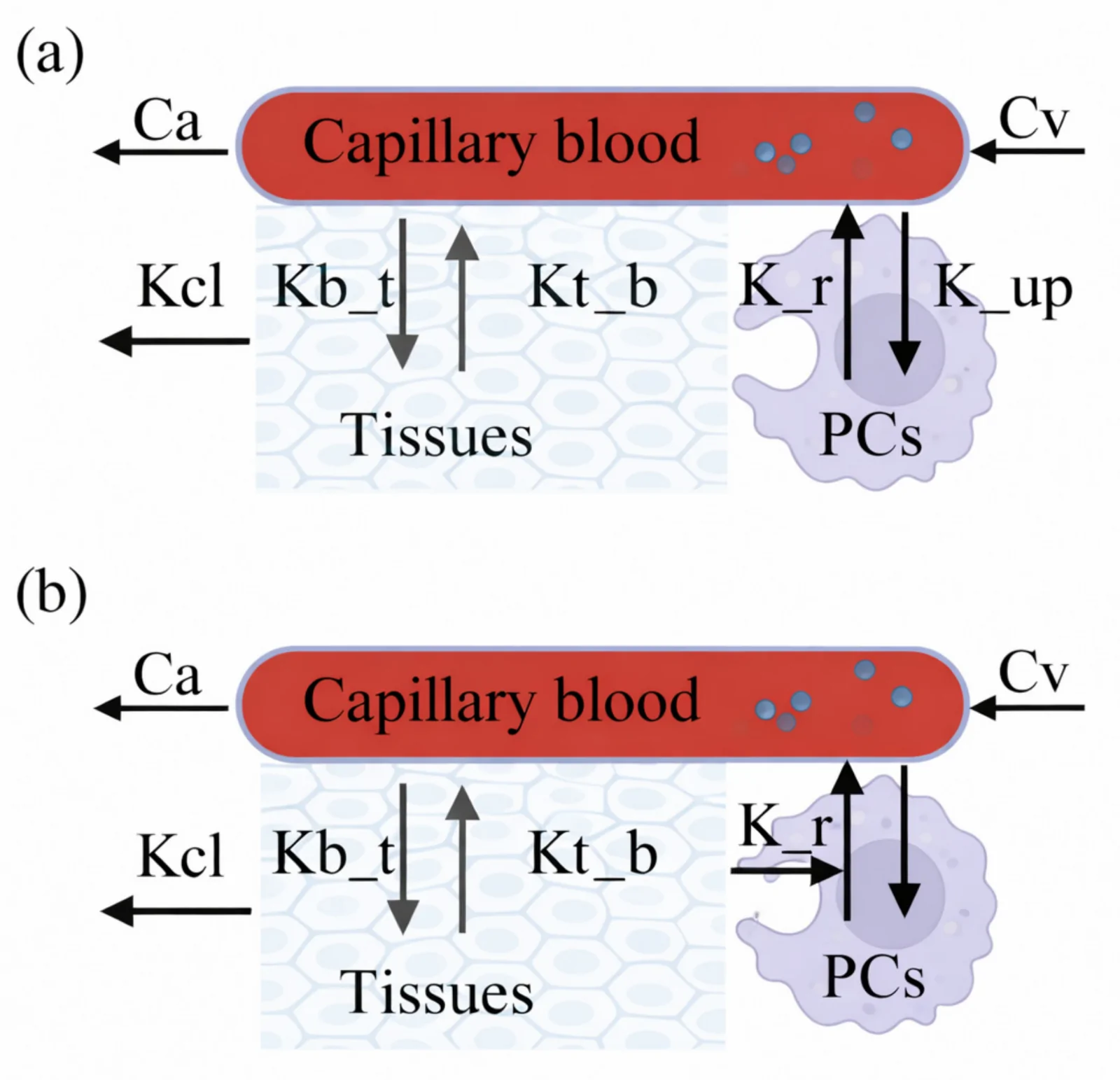
Schematic diagram of (**a**) extravascular route: DNCs extravasate from capillary blood into tissue commonly found in organs like the lung, GI tract, and kidney; (**b**) intravascular route: PCs, such as Kupffer cells, capture nanoparticles directly from blood, which is typical of the liver and spleen.

Figure 4 illustrates two pathways of DNC uptake by phagocytic cells (PCs) in systemic tissues: (a) Extravascular route—DNCs cross capillaries into the interstitium before phagocyte uptake, typically tissues with continuous or fenestrated capillaries (muscle, skin, heart, kidney, lung, GI tract) [29]. (b) Direct intravascular route—Phagocytes capture DNCs directly from blood, which is dominant in organs with discontinuous capillaries (liver, spleen, bone marrow). The dominant uptake route depends on tissue architecture (capillary wall type and pore size), DNC size (relative to endothelial gaps), and local immune surveillance (density/location of phagocytes) [29,30].

In the brain, the BBB presents a significant barrier due to tight junctions, low pinocytosis, and active efflux [31]. Effective CNS delivery typically requires receptor-mediated transcytosis or BBB-disruptive strategies. PBPK models incorporate the BBB as a permeability-limited barrier, using parameters such as the permeability–surface area product (PS_BBB_), efflux clearance, and receptor kinetics, thereby enabling quantitative prediction of brain DNC concentration profiles and guiding rational design.

While standard PBPK models treat each organ (including the brain) as a well-mixed compartment, more detailed structural approaches have been developed to capture spatial heterogeneity, particularly for the brain’s complex vasculature. Three-dimensional vascularized PBPK models incorporate anatomical data to mimic the in vivo geometry of cerebral blood vessels. Vascular heterogeneity and regional variations in cerebral blood flow can lead to uneven DNC distribution—features not captured by simplistic compartment models or 2D representations of the vasculature [32,33]. By reconstructing three-dimensional arterial, capillary, and venous networks from high-resolution imaging (MRI, CT), these models replicate in vivo anatomy and hemodynamics, enabling simulation of concentration gradients, regional perfusion differences, and localized BBB transport in a physiologically realistic manner [34,35]. Incorporating measured BBB properties and brain tissue kinetics into 3D frameworks further improves predictions of the spatial and temporal distribution of DNCs. Such anatomically realistic models are especially valuable for neurological diseases where BBB integrity or cerebral perfusion is altered, as they can capture disease-induced changes in delivery. Advances in bioprinting have even enabled in vitro 3D brain microvessel networks [36,37,38,39], and ML tools now assist in 3D model construction: ML algorithms can segment vasculature from imaging data, reconstruct continuous vascular networks, simulate blood flow dynamics, and preprocess images to reduce noise [40,41,42,43]. These integrated structural approaches greatly enhance the fidelity of PBPK models and support the rational design of brain-targeted DNCs.

Accurate modeling of BBB permeability is therefore critical for predicting DNC distribution in the brain. Approaches range from empirical QSAR correlations [44]—which relate molecular properties (lipophilicity, molecular size, etc.) to observed BBB penetration—to fully mechanistic PBPK models that simulate each step of ADME. ML techniques (neural networks, SVMs, and random forests) have also been trained to predict BBB permeability directly from molecular features [33]. Pathological conditions (e.g., neuroinflammation or neurodegenerative disease) can alter BBB integrity and transporter expression; emerging PBPK models account for such disease-specific changes by adjusting permeability coefficients, transporter levels, or including inflammation-mediated effects, thereby improving prediction accuracy for therapeutics under those conditions [45].

### 3.2. Challenges of Modeling CNS Physiological Barriers and Delivery Routes in PBPK

**Figure 5:**
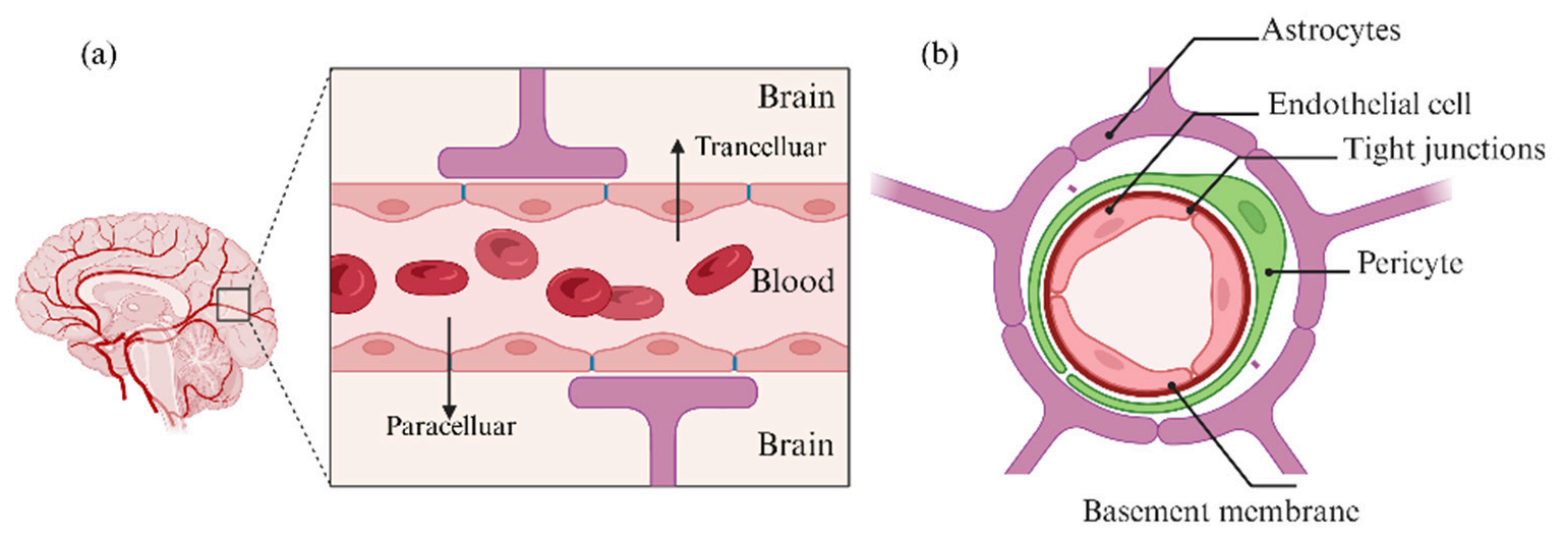
BBB transport routes that constrain ML–PBPK parameterization. (**a**) The paracellular pathway is effectively closed to most DNCs by tight-junction proteins (claudin-5, occludin, ZO-1), so its permeability term in PBPK models is typically fixed near zero in the healthy state but must be relaxed in disease states with documented junctional opening (stroke, glioma, neuroinflammation). The transcellular pathway dominates DNC entry and is partitioned into passive lipophilic diffusion, receptor-mediated transcytosis (e.g., transferrin, LRP1, insulin), and active efflux (P-gp, BCRP, MRPs); each branch contributes a separable ML-predictable parameter (PSeff, kRMT, Jeff) rather than a single parameter of global “BBB permeability.” (**b**) The associated cellular and mural elements—endothelial cells, pericytes, basement membranes, and astrocytic end-feet—define the surface area and accessory transporters that scale these parameters across species and disease states and are the geometric anchor for the imaging-informed 3D vascular reconstructions discussed in Section 3.2.

**Figure 6:**
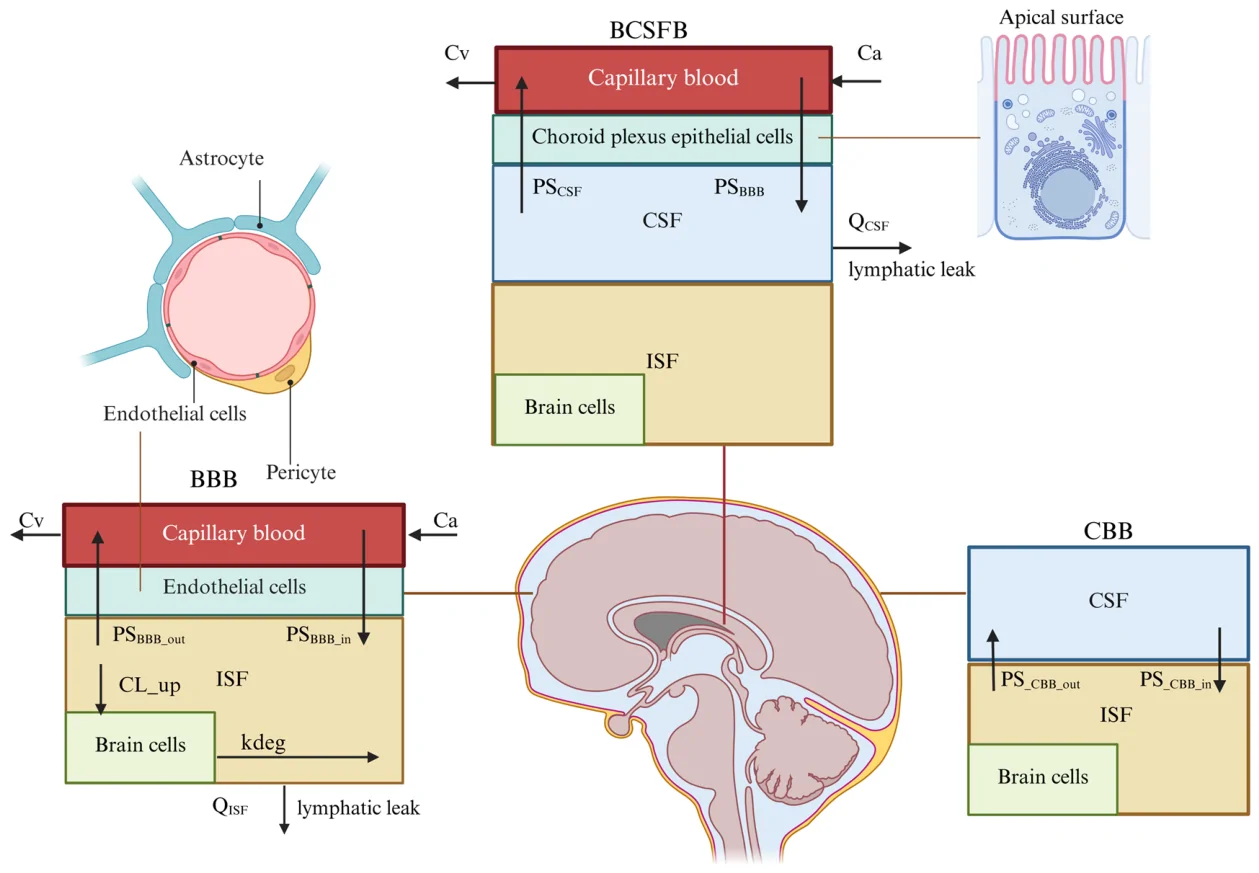
Schematic diagram of brain barrier structure supports quantitative PBPK modeling by visually organizing the anatomical interfaces and associated transport mechanisms critical to DNC distribution and clearance in the brain. C_a_ and C_v_ represent DNC concentration in arterial and venous blood; PSCSF and PS_BBB_ represent the transport through BCSFB and the BBB. PS_BBB_in_ represents the rate at which DNCs enter brain tissue from the bloodstream across the endothelial cells. PS_BBB_out_ describes the rate of efflux of DNCs from brain tissue back into the bloodstream (capillaries). PS_CBB_in_ and PS_CBB_out_ represent CSF-to-ISF influx and ISF-to-CSF efflux. In all three barrier units, red boxes denote capillary (blood) compartments, green boxes denote cellular barrier layers (vascular endothelial cells, choroid plexus epithelial cells, and brain cells), light-blue boxes denote cerebrospinal fluid (CSF), and tan boxes denote brain interstitial fluid (ISF). Horizontal arrows indicate arterial inflow (Ca) and venous outflow (Cv) of blood; paired vertical arrows indicate bidirectional permeability-surface-area (PS) transport across each barrier; and downward arrows labeled QCSF and QISF indicate lymphatic/CSF drainage (“lymphatic leak”).

The brain is protected not only by the BBB (Figure 5) but also by the blood–CSF barrier (BCSFB) [46] and the CSF–brain barrier (CBB) [47], each of which has distinct anatomy and transport characteristics (Figure 6). BBB transport occurs via paracellular (restricted) or transcellular (lipophilic or receptor-mediated) routes. PBPK models use parameters like permeability–surface area products (PS_BBB_, PS_CSF_, PS_CBB_) and advection–diffusion equations [23] to simulate directional exchange between blood, brain interstitial fluid, and CSF, including efflux and lymphatic clearance.

Incorporating these physiological barriers is essential for capturing the rate-limiting steps of brain uptake, thereby enabling the quantitative prediction of DNC distribution and guiding the design of CNS-targeted DNCs.

Beyond the barriers themselves, the route of drug administration profoundly influences DNCs’ absorption and distribution profiles (Table 3). The pharmacokinetic behavior of a DNC can vary widely depending on the route of administration (intravenous, oral, intranasal, etc.), and PBPK models must adapt to each scenario. Table 3 illustrates several common routes of administration and their impact on DNC pharmacokinetics. Intravenous (IV) injection delivers the full dose directly into circulation, providing immediate bioavailability and rapid systemic distribution. By contrast, with oral administration, the drug is subject to incomplete gut absorption and first-pass hepatic metabolism, greatly reducing the fraction of the dose that reaches the brain. Intranasal and intra-arterial routes can offer more direct brain delivery: intranasal sprays target the CNS via the olfactory and trigeminal neural pathways (bypassing the BBB to some extent), and intra-arterial injections result in high local drug concentrations in brain arteries. Intracerebral (e.g., intraparenchymal or intraventricular) administration bypasses all peripheral barriers by delivering DNCs directly into brain tissue or CSF, minimizing systemic exposure [48]. Transdermal patches provide yet another alternative, enabling slow, sustained systemic absorption through the skin [49]. Selection of the optimal route is critical for CNS drug targeting efficacy and safety. PBPK models account for these differences with route-specific sub-models: for oral dosing, modules for gastrointestinal dissolution, absorption, and hepatic first-pass extraction are included (with oral bioavailability influenced by drug solubility, stability, lipophilicity, molecular size, and efflux transporter activity such as P-glycoprotein [50]); for intranasal dosing, nasal mucosal absorption and direct nose-to-brain transport pathways are incorporated. IV models are simpler, as they focus mainly on distribution and elimination because absorption is not a factor, whereas transdermal models include skin permeation kinetics. By integrating such route-specific processes, PBPK models can quantitatively predict how different delivery strategies impact overall drug exposure in the brain.

Vascular heterogeneity and variable flow rates in the brain contribute to uneven DNC distribution, a feature not captured by static 2D models [32,33]. In contrast, 3D vasculature frameworks replicate in vivo anatomy and hemodynamics, allowing for the simulation of concentration gradients, regional perfusion, and BBB transport in a physiologically realistic manner [34,35]. High-resolution imaging (MRI, CT) enables detailed reconstruction of arteries, veins, and capillaries [51,52,53]. Incorporating BBB properties and biochemical data further improves the prediction of spatial and temporal DNC distribution.

Such models are especially valuable in neurological diseases, where BBB dysfunction and altered perfusion affect drug delivery. Advances in bioprinting [36,37,38,39] and ML further enhance 3D modeling: ML can segment vasculature from imaging, reconstruct continuous vascular networks, simulate blood flow dynamics, and preprocess imaging data to reduce noise [40,41,42,43]. These integrated approaches enhance the fidelity of PBPK models and support the rational design of brain-targeted DNCs.

### 3.3. Physicochemical Properties and Hemodynamic Challenges

The physicochemical properties of DNC play a pivotal role in their ability to cross the BBB and distribute within the CNS. Table 4 summarizes the major classes of DNC platforms and representative chemotherapeutic agents that have been explored for brain delivery. These include polymeric DNCs (e.g., dendrimers and polymeric micelles), liposomes, metallic and inorganic nanoparticles (e.g., iron oxide and gold), semiconductor quantum dots, and hydrogel nanoparticles (nanogels). Each DNC offers distinct advantages that can be leveraged to overcome the restrictive nature of the BBB. For example, polymer-based systems can be engineered for controlled and sustained drug release, have high drug-loading capacity, and can be functionalized with targeting ligands to engage receptor-mediated transport mechanisms; liposomes are biocompatible and versatile in encapsulating both hydrophilic and hydrophobic drugs; inorganic nanoparticles can provide magnetic or photothermal functionalities for guided delivery or theranostic applications. The table lists representative drugs across multiple disease categories, providing a concise reference for promising brain-targeted drug-delivery strategies beyond oncology.

Table 4 illustrates that agents of the same DNC class can yield markedly different brain exposure outcomes: for example, liposomes range from conventional formulations with minimal BBB penetration to PEGylated, transferrin-targeted variants that achieve measurable CNS uptake, while polymeric systems similarly span a wide range depending on size (50–300 nm), surface functionalization, and administration route. These within-class differences arise from variations in surface ligand density, hydrodynamic diameter, surface charge, and route of administration, making direct head-to-head comparison across the cited studies methodologically inappropriate without normalization of these parameters. To prioritize among classes for ML–PBPK modeling, three quantitative criteria are most informative, since their parameters dominate the predictive performance of PBPK models for brain delivery: (i) measured or predicted apparent BBB permeability (PSeff or Kp, uu, brain), (ii) reported plasma stability and circulation half-life, and (iii) reproducibility of size and surface charge across batches. Among the listed classes listed, PEGylated liposomes and polymeric nanoparticles functionalized with transferrin- or LRP1-targeting ligands have the most consistent quantitative datasets in the literature and are therefore the most tractable starting points for ML–PBPK calibration. Supporting this, published studies report K_0_, uu, brain values of 0.02–0.12 for PEGylated liposomes (100–150 nm, transferrin-conjugated) and plasma half-lives of 6–24 h [62,63,64], whereas transferrin-targeted PLGA nanoparticles achieve PS_tt_ estimates of 0.5–3.0 μL/min/g of brain with a batch-to-batch size reproducibility of ±10 nm [55,56,57,58,59,60,61]. Dendrimers and inorganic carriers, while versatile, currently have sparser in vivo brain-PK data and should be modeled with explicit uncertainty bounds.

Several additional platforms are being explored for brain-targeted delivery and could be incorporated into ML–PBPK frameworks in future work. Solid lipid nanoparticles (SLNs), and their second-generation counterparts, nanostructured lipid carriers (NLCs), provide a biocompatible solid-lipid matrix well suited to lipophilic agents; their physiological stability and amenability to ligand functionalization have been exploited to promote receptor-mediated transport across the BBB [73,74,75]. Cyclodextrin inclusion complexes use host–guest chemistry to improve the aqueous solubility and stability of poorly water-soluble CNS drugs, and—beyond their role as solubilizing excipients—can modulate BBB efflux transporters and be combined with lipidic or polymeric carriers to enhance CNS delivery [76]. Extracellular vehicles (EVs), including exosomes (typically 30–150 nm), are endogenous, cell-derived carriers characterized by low immunogenicity and an intrinsic capacity to traverse the BBB via endocytic and transcytotic routes, positioning them as a biological alternative to fully synthetic systems [77,78]. Integrating the distinct physicochemical and transport properties of these carriers into PBPK parameterization represents a natural extension of the modeling approaches reviewed here.

**Figure 7:**
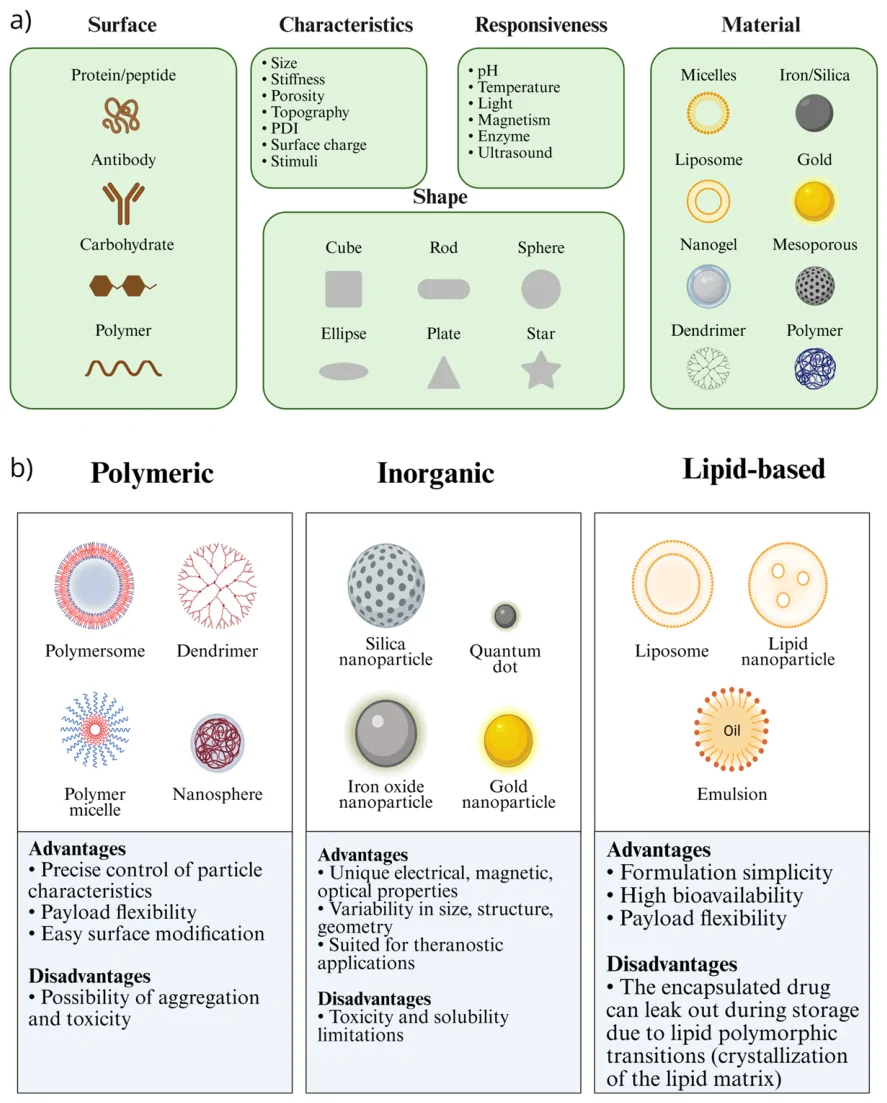
(**a**) Properties affecting DNCs’ brain drug delivery efficiency; (**b**) DNC categories.

In addition to the choice of DNC material, the specific design features of DNCs strongly influence their behavior in the bloodstream and at the BBB (Figure 7a). Surface characteristics are critical: the attachment of proteins, antibodies, or other targeting ligands to a DNC’s surface can facilitate receptor-mediated uptake into the brain, whereas hydrophilic polymer coatings (e.g., PEGylation) can provide a “stealth” effect that reduces opsonization and prolongs circulation time. The composition of the DNC core (whether lipidic, polymeric, or inorganic) and the particle’s shape (spherical vs. rod-like, etc.) also affect how it navigates the bloodstream and interacts with endothelial barriers. Many DNCs are engineered to be stimuli-responsive, changing their properties or releasing drugs in response to specific triggers such as pH, temperature, light, or magnetic fields—features that can be harnessed for targeted delivery. Figure 7b categorizes DNCs into three broad types—polymeric (e.g., dendrimers, polymeric micelles), inorganic (e.g., silica, gold, iron oxide nanoparticles), and lipid-based systems (liposomes, nanoemulsions) [79]—each with its own set of advantages and limitations in terms of functionalization potential, toxicity, solubility, and drug encapsulation efficiency. Empirical studies have elucidated several key trends: a high surface area (as in ultra-fine or porous nanoparticles) allows multivalent ligand conjugation for efficient receptor-mediated transport across the BBB [19]; the addition of “stealth” coatings like poly(ethylene glycol) (PEG) helps DNCs evade the mononuclear phagocyte system, increasing their plasma half-life [67] and thereby improving the likelihood of BBB penetration; surface charge and hydrodynamic diameter jointly modulate BBB transit, with weakly cationic carriers experiencing stronger electrostatic interaction with the anionic endothelial glycocalyx than strongly anionic carriers [80], and smaller DNCs (<~200 nm)—primarily if roughly spherical—generally exhibiting higher translocation efficiency across the BBB than larger ones [81].

Another important consideration is the protein corona that forms around DNCs in biological fluids. As a DNC circulates and eventually traverses the BBB, the composition of adsorbed proteins on its surface can change. Specific plasma proteins bound in the periphery may dissociate upon entering the brain microenvironment, while new proteins (present in CNS interstitial fluid) adsorb, thus altering the DNC’s bioidentity. This evolving protein corona can significantly affect DNC uptake and biodistribution: it may mask or reveal functional ligands and can influence recognition by cell-surface receptors. Studies have shown that some plasma proteins are selectively stripped off during BBB transit, potentially increasing nanoparticle stability and retention within the brain [82]. Incorporating species-specific corona formation and kinetics into PBPK models can enhance their predictive power and improve extrapolation from animal models to humans [83], by accounting for inter-species differences in protein binding that impact CNS delivery.

Despite the optimal design of DNC properties, hemodynamic factors in the bloodstream and microcirculation pose additional challenges for brain delivery. BBB endothelial cells are continuously exposed to shear stress from blood flow, and higher shear can reduce the adhesion and internalization of DNCs at the vessel wall [84]. Within microvessels, red blood cells tend to migrate toward the center of the lumen, creating a cell-free layer along the endothelium; this effect, together with rapid capillary transit times, limits the contact time between circulating DNCs and the BBB [85]. Consequently, many DNCs pass through cerebral capillaries without interacting significantly with the endothelium. Furthermore, once in systemic circulation, DNCs can be destabilized or eliminated by biological processes: enzymatic degradation can break down certain NCs or their payload, opsonins can tag them for clearance, and the mononuclear phagocyte system (e.g., liver and spleen macrophages) can rapidly sequester and remove them from circulation [86,87]. All of these factors can dramatically reduce the fraction of an administered dose that ultimately reaches the brain.

To overcome these flow-related barriers, researchers are pursuing a variety of strategies. One approach is to exploit or mimic endogenous transport pathways. For instance, DNCs can be “hitchhiked” on immune cells (such as monocytes or neutrophils) that naturally transmigrate across the BBB, allowing drugs to be effectively ferried into the brain via cell-mediated transcytosis [88]. Another strategy is to design stimuli-responsive DNCs that only release their drug payload or present their targeting ligands under specific conditions—for example, pH-sensitive or enzyme-sensitive particles that activate upon encountering the tumor microenvironment, or magnetically guided particles that concentrate in a target region under an external magnetic field [89]. Ligand-mediated targeting remains a cornerstone: functionalizing the nanoparticle surface with ligands (such as transferrin, lactoferrin, or insulin) that bind to receptors on the BBB can greatly enhance receptor-mediated endocytosis and transcytosis of DNCs [90]. To ensure these ligands remain attached in the dynamic flow environment, robust bioconjugation techniques such as click chemistry or biotin–avidin linkages are employed, thereby improving the stability of surface functionalization [91]. DNCs are also being engineered to bypass or inhibit efflux transporters; for example, modifying or cloaking drugs to evade P-gp recognition [76] can increase net BBB penetration. Physical and biochemical modulation of the BBB is another adjunct strategy; techniques such as focused ultrasound (often with microbubbles), photothermal heating, or osmotic/chemical BBB openers [92] can transiently increase BBB permeability, allowing more DNCs to cross. Finally, optimization of basic DNC design parameters (size, shape, surface charge, ligand density) is continually informed by in vivo studies to ensure the particles have maximal vascular residence time and adhesion potential under physiological flow conditions. By integrating these strategies, modern PBPK models can incorporate not just baseline biology but also interventional approaches to enhance drug delivery to the brain. Each interventional strategy requires specific additional parameters to be incorporated into the PBPK model structure. Cell-mediated transcytosis (e.g., hitchhiking on monocytes or neutrophils) requires the addition of a cellular carrier compartment with rate constants for DNC loading onto immune cells, transmigration kinetics across the BBB, and intracellular release. Stimuli-responsive DNCs necessitate conditional-release sub-models governed by local trigger concentrations (e.g., pH, H_2_O_2_, enzyme activity) or applied field intensity (e.g., magnetic field strength, ultrasound pressure amplitude), with release rate constants that switch between quiescent and activated states. Ligand-mediated targeting introduces receptor occupancy equations (kon, koff, receptor density) and endocytosis/transcytosis rate constants that scale with ligand valency and receptor expression levels. Efflux transporter inhibition can be modeled by adding a saturable efflux term (Vmax, Km) to the BBB sub-model and a competitive inhibition factor (Ki) when co-administered inhibitors are present. Physical BBB disruption (focused ultrasound, osmotic openers) is incorporated by making the PSₑⁱⁱ term time-dependent—briefly elevated during the disruption window and returning to baseline thereafter—with the magnitude and duration fitted to imaging or PK data. Explicitly parameterizing these interventions within the PBPK framework transforms the model from a passive descriptor of drug disposition into an active tool for optimizing delivery strategy, dose timing, and nanocarrier design.

### 3.4. PBPK Model Parameterization and Estimation

An integral aspect of PBPK modeling is defining and obtaining numerous parameters that describe anatomy, physiology, biochemistry, and drug-specific kinetics. One traditional metric of BBB penetration is the brain-to-blood concentration ratio (log BB); however, measuring the log BB in vivo is laborious and often yields limited, variable data points. Recent approaches that couple machine learning with PBPK modeling have shifted toward classification frameworks, identifying compounds as BBB-permeable (BBB+) or not (BBB−) rather than relying on continuous log BB values [93]. This strategy can streamline early screening of NCs by focusing on qualitative permeability categorization. Table 5 provides an overview of the key parameters commonly used in PBPK models, grouped by category, along with their definitions. These include anatomical parameters (describing the size and volumes of the body and organs), physiological parameters (describing blood flow rates and other systemic functions), biochemical parameters (enzyme and protein binding levels), chemical-specific parameters (drug-specific ADME rates), transport parameters (membrane permeability and transporter activities), and application-specific parameters (dosing regimen details). Collectively, these parameters define the model and can be tuned or validated to account for inter-individual variability (age, sex, genetics) and pathological states [94].

Table 6 outlines common methods for parameter estimation. QSAR models, for example, provide initial estimates of biological activity, toxicity, pharmacokinetics, and physicochemical properties (e.g., solubility, permeability) by linking chemical structure to these traits [95]. Integrating QSAR-derived properties into PBPK models improves prediction accuracy, accelerating drug development and safety evaluation [96].

Overall, constructing a robust PBPK model for brain drug delivery requires the integration of structural, physiological, and mechanistic data through careful parameterization. By combining detailed anatomical modeling, appropriate representation of CNS barriers, tailored DNC design considerations, and thorough parameter determination, conventional PBPK modeling approaches can provide a sound basis for predicting DNC disposition in the brain and guiding the development of more effective CNS-targeted therapies.

However, the challenges outlined above also highlight that many key PBPK parameters for brain-targeted DNCs are difficult to measure directly, are sparsely reported across studies, and often exhibit substantial inter-individual and inter-species variability. As a result, parameter uncertainty and identifiability issues can limit the predictive performance and translational utility of conventional PBPK models. These limitations have motivated the integration of machine learning with PBPK modeling to support parametric estimation and model refinement.

## 4. ML Integration for PBPK Parametric Estimation

To overcome these constraints, ML–PBPK workflows have been proposed in which ML approaches predict chemical-specific and system-specific parameters from molecular descriptors and experimental data, and then integrate them into mechanistic PBPK models. While experimental determination is time-consuming and costly, ML approaches can predict chemical-specific parameters from molecular descriptors and in vitro data, accelerating PBPK model development.

### 4.1. Workflow of ML–PBPK Modeling

ML–PBPK modeling typically follows three phases: (1) data collection and curation from databases and the literature, (2) feature extraction and ML training to predict PK parameters, and (3) integration into PBPK simulations to generate concentration–time profiles. This integration enables prediction for novel compounds, inter-species extrapolation, and personalized dosing while addressing chemical and physiological variability. ML–PBPK workflows predict human ADME parameters (Cmax, AUC, clearance) and integrate them into mechanistic PBPK models [97,98]. Advanced methods, such as neural ODEs [99], can model complete PK/PD profiles directly from early data, outperforming standard population-based approaches. Algorithms such as LightGBM, Support Vector Machine (SVM), decision trees, and random forests have been applied to predict PK parameters and BBB permeability, striking a balance between interpretability and robustness [5,16,80,100,101]. However, all these ML approaches rely heavily on feature engineering and suffer from redundant feature processing or over-fitting.

**Figure 8:**
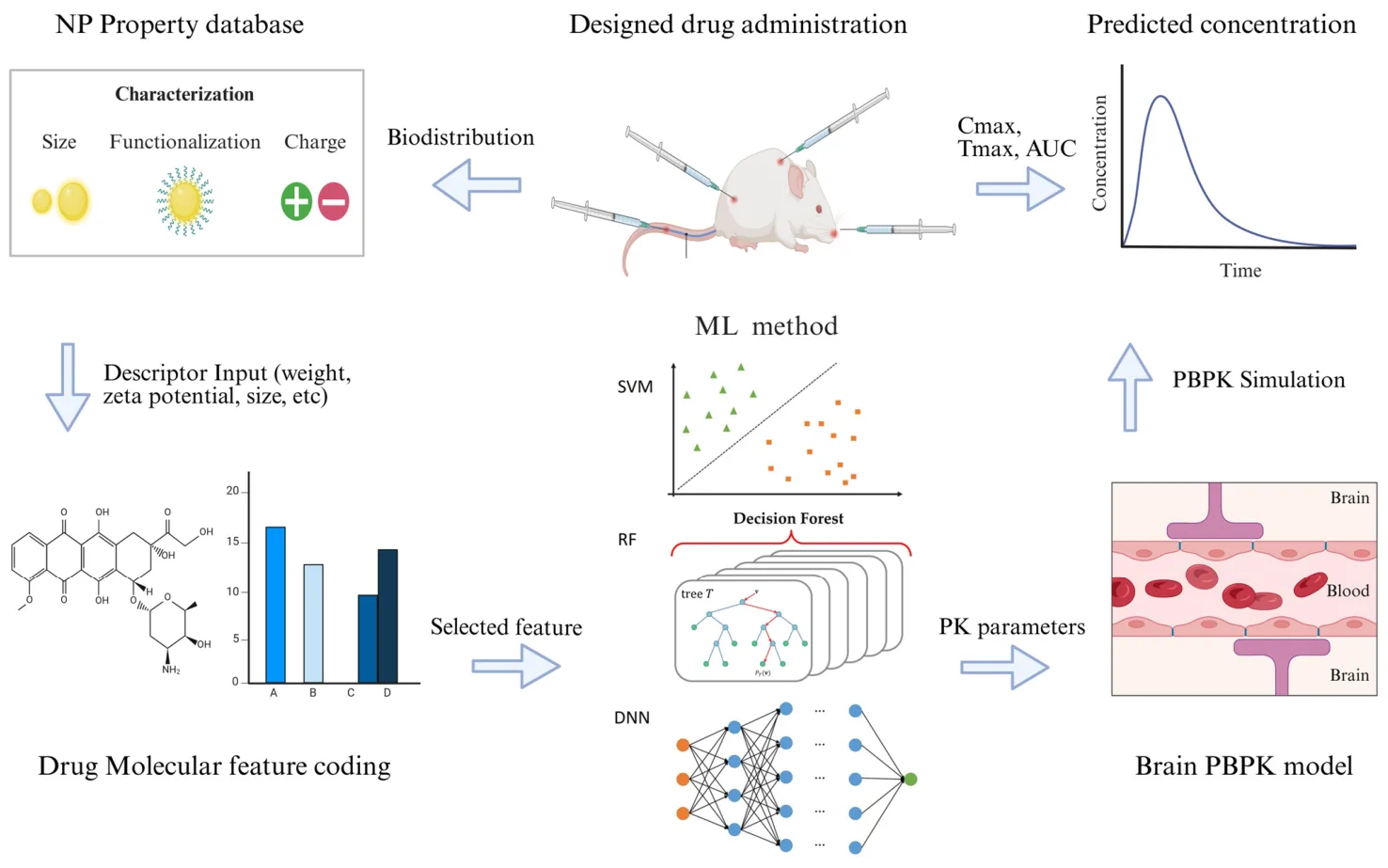
ML–PBPK modeling framework.

Figure 8 outlines the ML–PBPK workflow: collecting DNC physicochemical and biological data, extracting molecular features, selecting ML algorithms, validating models, and predicting concentration–time profiles. DNC properties (size, charge, functionalization) are compiled into databases, encoded as molecular descriptors, and used as input to ML models such as SVMs, random forests, or Deep Neural Networks (DNNs) to identify patterns and predict pharmacokinetics.

The data sources that feed ML–PBPK pipelines for brain delivery vary widely in scale, curation, and assay traceability, and these differences—rather than algorithm choice—often dominate predictive performance. PubChem (>100 million compounds) and DrugBank (~17,000 drug entries) supply broad chemical and target annotation but mix human, in vitro, and in silico provenance without uniform quality flags; FAERS provides post-marketing adverse-event signals that are useful for toxicodynamic anchoring but is not designed for quantitative PK modeling. For BBB-specific tasks, the curated logBB and Kp, uu, brain compilations behind Brainpeps, PK/DB, and the Wang/Adenot logBB sets contain at most a few thousand unique compounds, are dominated by small lipophilic molecules, and under-represent biologics, polymeric carriers, and lipid nanoparticles. Many of the same logBB measurements are recycled across these resources, so reported test-set accuracies often overstate true generalizability. Table 7 shows a tiered set of resources whose suitability depends on the specific brain-delivery context, the chemical space of the candidate carrier, and whether the downstream PBPK model needs absolute concentrations or only relative ranking.

Feature selection reduces redundant descriptors to enhance model accuracy, interpretability, and computational efficiency [113]. Methods include Scikit-learn’s “Select from model,” Recursive Feature Elimination (RFE), Information Gain ranking, heuristic approaches, and hybrid Genetic Algorithm–Partial Least Squares (GA-PLS) techniques [114]. RFE and GA-PLS have successfully identified key descriptors—such as polar surface area, molecular rugosity, and hydrophobicity—that strongly influence BBB penetration. Combining dimensionality reduction with algorithms like SVM, random forest, or Artificial Neural Network (ANN) enhances model performance, particularly for imbalanced datasets, enabling accurate prediction of BBB permeability (e.g., LogBB) and pharmacokinetic parameters.

An SVM model trained on 150 compounds achieved an accuracy of 82% for BBB+ prediction [44]. Dimensionality-reduction techniques, such as RFE applied to 199 descriptors, identified 37 key features affecting BBB penetration (e.g., polar surface area, molecular rugosity, hydrophobicity) [115]. Combining SVM with synthetic SMOTE and MACCS fingerprints on 2358 compounds improved classification of imbalanced datasets (training/test accuracies: 0.975/0.907) [116]. Other studies using Multiple Linear Regression (MLR), SVM, and ANN on 320 compounds accurately predicted LogBB [117]. Heuristic feature selection quickly identifies missing, insignificant, or highly correlated descriptors, producing results 2–5× faster than other methods [118,119]. Hybrid GA-PLS approaches reduce descriptor number and enhance model predictive power, with as few as seven descriptors yielding robust, interpretable ML models [113].

Supervised ML models correlate selected features with outcomes such as DNC tissue distribution or PK parameters [114]. Training involves minimizing prediction errors using cross-validation and fine-tuning hyperparameters or PBPK physiological inputs. The trained ML–PBPK model simulates DNC concentration–time profiles across tissues under various dosing scenarios.

Model accuracy is assessed using R^2^, Root Mean Square Error (RMSE), and twofold error scores. R^2^ measures the proportion of variance explained, RMSE quantifies prediction errors, and the twofold error evaluates predictions within acceptable industry ranges for AUC. Fivefold cross-validation and 95% confidence intervals ensure generalizability and robustness of predictions. Additionally, the Matthews correlation coefficient (MCC) was calculated to assess the classification performance.

(1) $$Q=\frac{\mathrm{TP}+\mathrm{TN}}{\mathrm{TP}+\mathrm{FN}+\mathrm{TN}+\mathrm{FP}}$$

where TP presents the number of true positives, TN the true negatives, FN the false negatives, and FP the false positives:

(2) $$\mathrm{SP}=\frac{\mathrm{TN}}{\mathrm{TN}+\mathrm{FP}}$$

(3) $$\mathrm{SE}=\frac{\mathrm{TP}}{\mathrm{TP}+\mathrm{FN}}$$

(4) $$\mathrm{MCC}=\frac{\mathrm{TP}\times \mathrm{TN}-\mathrm{FP}\times \mathrm{FN}}{\sqrt{\left(\mathrm{TP}+\mathrm{FP}\right)\left(\mathrm{TP}+\mathrm{FN}\right)\left(\mathrm{TN}+\mathrm{FP}\right)\left(\mathrm{TN}+\mathrm{FN}\right)}}$$

By combining ML with PBPK modeling, researchers can achieve more accurate and individualized predictions of DNC distribution in the brain, ultimately improving treatment outcomes for neurological disorders.

### 4.2. Conventional PBPK vs. ML–PBPK for BBB Prediction

Table 8 summarizes conventional PBPK models for brain permeability. While fairly accurate, PBPK models require extensive experimental data, considerable time and human effort, and may perform poorly for poorly characterized drugs or enzymes [120].

Table 9 highlights ML approaches, which show strong potential for predicting BBB permeability and related PK parameters. Classical methods, such as SVM and PLS-DA, achieved accuracies greater than 0.90 [108,113], with refinements like resampling improving performance (SVM: 0.938) [116]. Nonlinear kernel methods (e.g., LS-SVM) model partition coefficients precisely (RMSE ~0.02) [118], while hybrid approaches combining physicochemical descriptors and feature selection improve interpretability, though sometimes inconsistently (AUC ~0.6) [125].

Deep learning models, including recurrent and fully connected neural networks, achieve high prediction accuracy (0.965–0.981) [126,127]. Ensemble frameworks (RF, GBM) also perform strongly (AUC ~ 0.97) [128,129]. Graph neural networks show moderate gains (AUC ~ 0.72), likely limited by dataset size [122]. Common challenges include varying endpoint definitions, modest dataset sizes, limited external validation, and lack of integration with physiological PBPK constraints. Translational impact requires standardized benchmarks, PBPK integration, and cross-species validation [130,131]

**Table 9:** Performance comparison of selected reported BBB permeability prediction models.

Application Area	Modeling Method	Performance
**Brain PK parameter** [14]	SVM, RF, XGB, GBM	AUC 0.65
**BBB permeability, permeation** [108]	PLS-DA	ACC 0.910
**pKa** [109]	Linear regression	Standard deviation (SD) error 0.246
**Brain plasma partition coefficient** [132]	LMEM	MAE 0.197
**Log BB** [113]	SVM	RMSE (0.118)
**BBB permeability** [116]	MD + SVM + no resampling	ACC 0.938
**Brain plasma partition coefficient** [118]	Least-squares SVM	RMSE (0.02)
**BBB permeability, CNS permeability** [130]	SVM	ACC 0.815
**BBB permeability** [111]	Bayesian	ACC 0.95
**BBB permeability, penetration** [133]	NHD+NHA	ACC 0.748
**BBB permeability** [128]	Graph neural network	AUC-ROC (72%)
**BBB permeability** [134]	Ensemble models	AUC 0.966
**BBB permeability** [126]	Recurrent neural network (RNN)	ACC 0.965
**BBB permeability** [111]	RF	ACC 0.947
**BBB permeability** [125]	RF	ACC 0.870
**Brain plasma partition coefficient** [135]	DT, RF	RMSE (0.0289)
**Brain plasma partition coefficient** [136]	SVM-RFE	AUC-ROC 0.6
**Log BB** [129]	LightGBM	MSE 0.36
**BBB permeability** [131]	DeepB	ACC 0.74
**BBB permeability** [137]	ANN	RMSE 0.956
**BBB permeability** [138]	SVM	ACC 0.968
**Logpp** [139]	LightGBM	R 0.84
**BBB permeability** [127]	DNN	ACC 0.9807
**BBB permeability** [140]	RF, XGB, XFT	ACC 0.978
**Brain PK parameter** [141]	DNN	RMSE 0.74
**LogBB** [142]	ANN	RMSE 0.236
**BBB permeability** [143]	SVM	ACC 0.975

**Figure 9:**
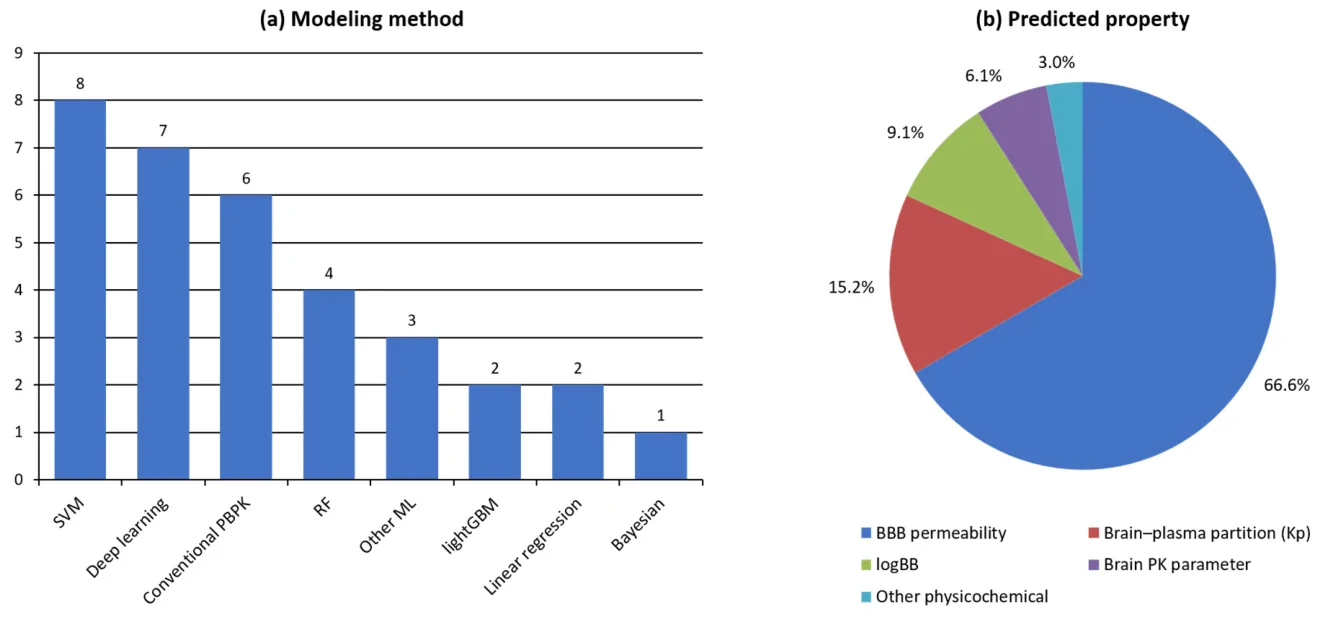
Distribution of the 33 studies included in the review: (**a**) modeling method; (**b**) predicted property.

Table 10 summarizes representative ML methods applied to PBPK and BBB-permeability problems alongside the dataset sizes and validation strategies on which their reported metrics depend, since headline values such as AUC, R^2^ and RMSE are only interpretable in that context. In most of the studies reviewed, models were trained on fewer than a few thousand compounds, with logBB and BBB-classification benchmarks typically containing 300–2000 molecules and many proprietary PK datasets fewer than 500. SVM models reach AUC ≈ 0.92–0.98 and accuracies above 0.95 on small, well-curated logBB sets, but these values are inflated relative to genuine generalization because the benchmarks are chemically narrow and frequently evaluated by random rather than scaffold or temporal splits. RF and GBM tolerate heterogeneous descriptors and modest sample sizes, yet their reported R^2^ values of 0.7–0.9 should be read as upper bounds because cross-validated estimates routinely drop by 0.10–0.20 when external test sets contain novel chemotypes [144,145,146]. DNNs achieve the lowest RMSE on time-resolved PK profiles when training data exceed a few thousand observations, but with the small per-tissue datasets typical of brain PBPK, they are prone to overfitting and produce metric improvements that are not always statistically distinguishable from those of simpler ensembles. The reported metrics therefore reflect both algorithmic capacity and data design, and direct comparison across studies is only meaningful when sample size, train/test partitioning and applicability domain are matched. Figure 9 quantifies this landscape: across the corpus reviewed in this study, RF and GBM-family models account for roughly one-third of reported ML–PBPK applications, classical SVM/regression for another quarter, and deep architectures (DNN, GNN, LSTM) for less than one-fifth, with the remainder consisting of hybrid or physics-informed approaches. The skew toward tree-based methods reflects the small, tabular, descriptor-driven datasets that dominate brain-PBPK research rather than any inherent superiority of those methods, and the relatively low representation of deep learning is consistent with the data-scarcity and interpretability constraints discussed above. Figure 9 should therefore be read as a map of current practice and their biases—not as a ranking of method performance—and it supports the recommendation in Section 6 to expand standardized, sufficiently large brain-PK datasets before drawing strong conclusions about which ML family is best suited to ML–PBPK integration.

### 4.3. ML–PBPK Advantages and Limitations

Compared with conventional PBPK models, ML–PBPK offers several advantages: (1) inductive capability to detect previously unseen patterns, (2) reduced need for human intervention for model updates, (3) rapid prediction across diverse tasks given sufficient feature data, and (4) applicability to post-market monitoring, where limited data may reduce PBPK accuracy. Its limitations include: (1) the need for large datasets and computational resources, with challenging interpretation for complex or nonlinear models, and (2) reliance on extensive drug-specific information, such as targets, interactions, and side effects [148].

A balanced view of ML–PBPK requires acknowledging where the approach has demonstrably failed or under-performed, since the literature is dominated by positive results that may overstate real-world utility. Three failure modes recur. First, generalization collapses when models trained on chemically narrow CNS-permeability sets are applied to new chemotypes: reported AUCs of 0.95+ on benchmarks such as the curated logBB datasets routinely fall below 0.75 on prospective external compounds [145,149], and several published QSAR-PBPK pipelines have produced negative R^2^ on temporal hold-outs, indicating a performance worse than predicting the dataset mean. Second, ML-augmented PBPK predictions for nanocarriers have repeatedly missed observed brain exposure by 5- to 20-fold when training data did not include the specific surface chemistry, size or protein-corona conditions of the test particle [150]; case studies of PEGylated and ligand-functionalized liposomes show that small changes in opsonization parameters can flip a model from accurately predicting brain Kp, uu to mis-ordering the ranking of candidate carriers [151]. Third, hybrid models that learn residuals on top of a mechanistic PBPK skeleton can fit training data tightly while violating mass balance or producing physiologically implausible tissue concentrations once driven outside the calibration window, a behavior reported for several deep learning–PBPK couplings [152] and one of the principal reasons regulators currently require mechanistic interpretability alongside any ML component. These failure cases do not invalidate the approach, but they delimit its current credible scope: ML–PBPK is most reliable for interpolation within well-characterized chemical and physiological ranges, and least reliable for extrapolation to novel nanocarriers, novel disease states, or pediatric and geriatric populations underrepresented in training data.

As ML-enhanced PBPK modeling becomes more sophisticated, its successful deployment increasingly depends on the capabilities of available software tools. However, current PBPK platforms vary widely in their ability to support DNC-specific parameters, detailed CNS physiology, and machine learning integration. Understanding the strengths and limitations of these software tools is critical to advancing ML–PBPK modeling into practical and regulatory settings. The following section outlines the key features and challenges of widely used PBPK simulation platforms.

## 5. Commercial Software: Challenges and Future Directions

Commercial PBPK software packages continue to play a vital role in drug development by providing user-friendly platforms, regulatory alignment, and broad simulation capabilities for ADME modeling. Yet when applied to brain-targeted therapies—especially those involving DNCs—these platforms often fall short due to simplified assumptions, limited ML integration, and limited mechanistic depth. Key gaps include: incomplete modeling of the blood–brain barrier and CNS-specific distribution routes, limited accommodation of DNC-specific kinetics (e.g., opsonization, protein corona dynamics, immune clearance), minimal support for importing ML-predicted parameters or linking to data-driven modules, and high computational overhead for complex models, often requiring advanced expertise to operate.

### 5.1. PK Modeling Software and Open Challenges

To better understand these challenges, Table 11 summarizes seven commonly used PBPK platforms, evaluating their primary applications and current limitations in modeling CNS-targeted drug delivery, particularly for ML-enhanced nanomedicine applications.

Table 11 reveals a clear stratification of fitness for brain-PBPK and ML–PBPK applications rather than a uniform set of equivalent options. Among commercial platforms, GastroPlus X.2 and Simcyp Simulator V25 provide the most mature CNS modules and the strongest regulatory acceptance, but both rely on closed parameter libraries and offer only limited hooks for importing externally trained ML models, so users typically have to choose between regulatory familiarity and ML extensibility. PK-Sim/MoBi v12 (Open Systems Pharmacology) is currently the most flexible commercial-quality option for ML coupling because its open source code and Matlab 2025a/R 4.6.1/Python 3.14.6 interfaces enable surrogate or residual ML modules to be wired directly into the ODE system, but its native DNC and BBB representations are still less detailed than those of Simcyp Simulator V25. Open-source alternatives such as Piranac (v25.7.1), BioGears (v7.3.2) or pure SBML 5.9.1/SBGN 2.0 pipelines support arbitrary ML integration and reproducibility but lack validated CNS physiological priors and have no formal regulatory standing, which limits their use beyond research. On the data side, public repositories such as ChEMBL, DrugBank, PubChem and the BBB-permeability subsets of Therapeutics Data Commons differ markedly in size, curation depth and chemical coverage: ChEMBL and PubChem dominate by volume but contain heterogeneous assay conditions that require harmonization before training, DrugBank offers cleaner but smaller drug-centric records, and TDC provides pre-split benchmarks that are convenient for ML but small enough to over-fit. None of the publicly available resources currently provides a sufficient number of high-quality, paired in vivo brain-exposure and DNC-physicochemistry records to train deep models from scratch. Practically, this means that for regulated brain-PBPK applications, Simcyp V25or PK-Sim plus GastroPlus X.2 targeted Kp, uu or Kp data from CNS-specific datasets remain the most defensible combination, while pure ML–PBPK research benefits from PK-Sim or open-source pipelines coupled with TDC-derived benchmarks supplemented by curated data from the literature. Future work in the field is unlikely to be limited by algorithm choice; it will be limited by the availability of standardized, openly licensed brain-PK and DNC datasets at a scale that current databases do not yet provide.

### 5.2. Emerging Strategies for Overcoming Pbpk Modeling Barriers in Brain-Targeted Nanomedicine

As limitations in current PBPK platforms become more evident—particularly for brain-targeted nanomedicine—strategies intended to overcome these barriers by integrating advanced modeling techniques, richer biological inputs, and machine learning-driven insights are emerging. These developments fall into three synergistic areas: mechanistic enhancements, data-driven parameter estimation, and personalized modeling approaches.

#### 5.2.1. Mechanistic Enhancements for CNS-Relevant Modeling

A major challenge in modeling DNC delivery to the brain lies in accurately representing biological processes such as opsonization, reticuloendothelial system (RES) clearance, endothelial uptake, and transcytosis. Conventional PBPK models often treat these processes as generic permeability terms. Still, new research emphasizes incorporating explicit DNC-specific interactions, including: (1) protein corona evolution, which alters nanoparticle surface identity during circulation and crossing the BBB [160]; (2) endocytosis and ligand–receptor kinetics, particularly for targeted NCs exploiting transferrin or insulin pathways [3,161,162,163]; and (3) size-, shape-, and charge-dependent biodistribution, which affects clearance, tissue uptake, and retention.

Recent modeling approaches integrate these mechanisms into PBPK frameworks, often with support from machine learning algorithms that estimate parameter values from experimental or high-throughput screening data [164,165]. Additionally, simulation fidelity is being improved through the use of imaging-calibrated models: techniques such as PET, MRI, and microdialysis now provide spatiotemporal concentration maps of drugs and DNCs in the brain [166], which inform and validate dynamic PBPK predictions.

#### 5.2.2. Data-Driven Modeling and ML Surrogates

ML models are increasingly used to create surrogate models that replicate complex PBPK outputs without solving large ODE systems, enabling faster simulations. These ML surrogates—often trained using neural networks or gradient boosting—can infer difficult-to-measure parameters such as BBB permeability, cellular uptake rates, and region-specific brain distributions from structural and physicochemical inputs [164,165]. Moreover, ML–PBPK workflows enable parameter tuning and uncertainty quantification, particularly when experimental data are sparse or noisy. For example, Bayesian networks can incorporate prior biological knowledge, while feature selection methods (e.g., GA-PLS, RFE) extract high-impact descriptors that influence brain uptake, facilitating more accurate and interpretable models [155,156].

#### 5.2.3. Toward Personalized “Virtual Twin” PBPK Models

The ultimate goal of next-generation PBPK modeling is personalized prediction. “Virtual twin” models integrate patient-specific variables—demographics, genotype, imaging biomarkers, disease state—to simulate individualized drug kinetics in the brain [167]. These models can inform precision dosing strategies, especially for vulnerable populations (e.g., pediatric, geriatric, neurodegenerative). Realizing this vision will require (i) open-source modeling platforms that support reproducible PBPK workflows, (ii) standardized ML benchmarks for cross-species and cross-disease validation, and (iii) prospective clinical datasets that link brain pharmacokinetics to measurable imaging biomarkers [168]. Together, these strategies pave the way for ML-integrated PBPK models that are not only more predictive but also more adaptable to the real-world complexity of CNS drug delivery.

## 6. Conclusions

Across the studies surveyed this review, three convergent findings emerge that are obscured when ML–PBPK is described only in aspirational terms. First, the predictive limit of brain-PK ML models is set primarily by the upstream data—the small size, narrow chemical space, and recycled provenance of public BBB datasets—rather than by algorithm choice; reported AUC and RMSE values should therefore be interpreted alongside dataset size, validation strategy, and the chemical similarity between training and test compounds. Second, the most defensible role for ML in PBPK is as a constrained surrogate for difficult sub-models (e.g., transporter kinetics, BBB permeability) rather than as a global replacement for mechanistic structure, because mechanism-anchored hybrids degrade more smoothly outside the training distribution than purely empirical regressors. Third, the field still lacks a shared reporting standard for negative results, prospective validation, and external test sets, which limits cross-study comparability. ML techniques, particularly deep learning, have demonstrated their ability to handle the nonlinear, high-dimensional data inherent in brain pharmacokinetics, enabling more accurate and personalized predictions of brain ADME. By integrating diverse data sources, including omics data, imaging, and patient-specific variables, ML–PBPK models can streamline drug development and inform personalized treatment strategies. Importantly, PBPK outputs can generate novel parameters for ML algorithms, providing mechanistic insights while improving predictive power. However, the limited interpretability of the black-box models remains a barrier, underscoring the need for inherently explainable approaches. Building reliable PBPK databases, expanding mechanistic details, and advancing interpretable ML methods will be essential. Together, these innovations position ML–PBPK integration to transform CNS drug development and accelerate precision brain therapeutics. We close with two caveats that research in this field has underemphasized so far. (i) Failure modes—dataset shift across species and disease states, silent extrapolation outside the convex hull of training descriptors, and overfitting to highly correlated logBB measurements—are widespread but rarely reported, and they can produce confidently wrong predictions for novel DNC chemistries. Routine reporting of applicability domain, prospective in vivo checks, and head-to-head benchmarks against simple mechanistic baselines should become a baseline expectation rather than an option. (ii) Generative AI tools, including large language models, are increasingly used during the literature curation, code generation, and manuscript drafting steps of ML–PBPK studies. While such use is not inherently problematic, authors should disclose where and how these tools were used, retain human accountability for every claim and citation, and not present unverified machine-generated text as primary scientific reasoning. Adoption of these standards will be at least as important as algorithmic progress in determining whether ML–PBPK becomes a trustworthy tool for CNS drug development.

## Figures and Tables

**Table 1:** Contributions and limitations of ML–PBPK review studies.

Review Title	Contribution	Limitations	Our Contribution
**Machine learning and in physiologically based pharmacokinetic modeling** [15]	It discusses the potential of neural ordinary differential equations (neural-ODE), a novel DL technique, to directly predict time-series PK profiles and its future application in PBPK modeling.	Details on validation metrics like R^2^, RMSE, etc., are provided for some models, but a systematic assessment of prediction accuracy across different parameter categories is lacking.	We provide a detailed performance evaluation and assessment for each case study.
**A decade of machine learning-based predictive models for humans pharmacokinetics: Advances and challenges** [16]	It reviews ML models with descriptors to predict PK parameters. Mordred and PaDEL with R^2^ > 0.87 for volume of distribution prediction; PaDEL and linear regression with 59% of drugs within twofold error; R^2^ of 0.532 for fraction unbound to plasma protein prediction; and Mordred, PaDEL, and MOE with RF and R^2^ > 0.87 for clearance prediction.	While some descriptor importance is discussed, the review does not evaluate the interpretability of the different modeling techniques used.	We assess the strengths and weaknesses of current ML–PBPK models for brain drug delivery. We provide insights into the most promising approaches while also pointing out gaps in knowledge or areas where models have failed to deliver accurate predictions.
**Review: Role of Model-Informed Drug Development Approaches in the Lifecycle of Drug Development and Regulatory Decision-Making** [17]	It summarizes how model-informed drug development (MIDD) like population PK modeling, exposure-response analysis, and disease progression models have supported regulatory evaluations and decisions according to the FDA’s experiences and perspectives.	Case studies and examples discussed are limited to a select few therapeutic areas, like oncology. Broader representation could have strengthened some points.	We showcase market applications of ML–PBPK models in drug discovery and development for neurological and psychiatric disorders with examples to predict brain drug concentrations and optimize dosage for safe and efficient brain-targeted therapies.
**Review of QSAR Models and Software Tools for predicting Biokinetic Properties** [18]	It discusses descriptors for further work, and outlines needed future research—advancing the application of in silico methods for ADME assessment.	While commonly used descriptors are discussed, a more systematic analysis of the most influential/predictive descriptors is lacking.	We discuss the selection of relevant molecular descriptors for brain modeling, emphasizing the role of feature selection algorithms in identifying the most predictive features.
**Artificial intelligence and machine learning disciplines with the potential to improve the nanotoxicology and nanomedicine fields: a comprehensive review** [19]	It discusses how AI/ML–PBPK modeling, nano-QSAR modeling, and toxicogenomics can be applied to analyze and interpret large toxicology datasets, predict nanocarrier behavior and toxicity, and study the genetic basis of toxicity.	It is completely based on published literature, and there is a chance of bias in studies included depending on the research methodology and databases used.	We discuss the scarcity of high-quality experimental data for brain pharmacokinetics, which is crucial for training robust ML models. We explore how ML techniques can compensate for or mitigate these data limitations.
**An Artificial Intelligence Framework for Optimal Drug Design** [20]	It establishes a proof-of-concept for a fully integrated ML framework to optimize drug properties like brain exposure through de novo molecular design guided by predictive models.	Only 21 compounds had human brain PK data, which is insufficient. Models had high uncertainty when fitted to human data.	Case study with a large quantity of human in vitro datasets for training.
**Engineering precision nanoparticles for drug delivery** [21]	It highlights how nanocarrier design can improve general efficacy while enabling precision medicine applications, with a focus on overcoming heterogeneous biological barriers through rational engineering approaches.	There is a significant gap between animal studies and human clinical applications, primarily due to differences in physiology and pathology between species, which affect the behavior and functionality of nanomedicines.	A discussion on the dataset of different species is included in this review, as well as the method of cross-species modeling.
**Artificial Intelligence and Machine Learning in Computational Nanotoxicology: Unlocking and Empowering Nanomedicine** [22]	It provides an overview of how ML is applied in computational nanotoxicology to enhance modeling, predict toxicity, reduce animal testing, and ultimately help translate nanomedicines to the clinic in a safer manner.	It does not systematically assess or rank the different computational methods discussed in terms of their current predictive capabilities and limitations, which could help readers understand the state of the art.	We compare traditional PBPK with ML-enhanced models, and discuss the advantages and limitations of each, particularly in handling the complexities of the BBB and other PK processes.

**Table 2:** Inclusion and exclusion criteria for study inclusion.

Category	Criterion
Databases searched	PubMed, Web of Science, Scopus
Search string	“Machine Learning” “Deep Learning” OR “Artificial Intelligence” OR “Neural Network” OR “Random Forest” OR “Support Vector” OR “Gradient Boosting” AND “Physiologically Based Pharmacokinetic” OR “PBPK” OR “ Pharmacokinetic Model” OR “Compartmental Model” AND “Blood-brain Barrier” OR “BBB” OR “Central Nervous System” OR “CNS”, Refined by publication years 2008–2025
Publication period	January 2008–December 2025
Study type	Peer-reviewed primary literature
Inclusion—scope	Combined at least one ML algorithm with a PBPK or compartmental brain-PK model for a CNS- or BBB-relevant application
Inclusion—ML specificity	Must specify an actual ML algorithm (not a generic modeling approach)
Inclusion—performance reporting	Must report at least one quantitative metric (AUC, RMSE, R^2^, or equivalent)
Inclusion—PBPK linkage	ML output must connect to a PBPK or compartmental brain-PK component; stand-alone QSAR studies excluded
Inclusion—data transparency	Must disclose a traceable data source or share code
Exclusion	Conference abstracts, editorials, ML studies without PBPK linkage, mechanistic PBPK studies without ML
Data extracted per study	Validation strategy, dataset size, class-imbalance handling, data origin (in vitro/in vivo/clinical/in silico), and whether tested on independent or prospective data

**Table 3:** Adaptation of the PBPK model structure by route of administration for brain-targeted delivery.

RoutRoutee	PBPK StructuPBPK Structural Modificationral Modification	KeKey Model Parametersy Model Parameters	Brain AcBrain Access Pathwaycess Pathway	Special CSpecial Considerationsonsiderations
Intravenous (IV)	[Direct input] No absorption sub-model; dose enters central blood compartment instantaneously; standard multi-compartment structure	Vd, CL, fu (unbound fraction), protein binding, tissue partition coefficients (Kp)	Systemic circulation → BBB (passive/transporter-mediated)	Reference route; F = 1; baseline for BBB permeability estimation
Oral	[Absorption sub-model] GI tract compartments (stomach, small intestine); first-pass hepatic extraction module added upstream of systemic circulation	ka, Fg (gut wall), Fh (hepatic), dissolution rate, Peff (intestinal permeability), P-gp efflux	Gut → portal vein → liver (first-pass) → systemic → BBB	Substantially reduced brain exposure; lipophilicity and P-gp efflux must be parameterized
Intranasal	[Partial BBB bypass] Nasal mucosa compartment with dual output: systemic absorption + direct nose-to-brain transfer via olfactory/trigeminal pathways	Pnasal, kolf (olfactory transfer rate), mucociliary clearance, CSF input rate	Nasal epithelium → olfactory nerve → olfactory bulb/CSF; concurrent systemic route	Two-pathway model required; olfactory direct transfer bypasses first-pass and BBB
Intra-arterial	[Direct arterial input] Dose injected into arterial compartment proximal to brain; high first-pass cerebral extraction modeled before systemic dilution	Qbrain (cerebral blood flow), Ebrain (extraction fraction), PS_BBB_, arterial–venous concentration difference	Arterial blood → cerebral capillaries → brain parenchyma (high local concentration)	Avoids systemic dilution; brain extraction modeled as single-pass
Intracerebral/Intraventricular	[Local CNS input] Dose applied directly to brain tissue or CSF; peripheral barriers bypassed; CSF flow and ISF diffusion govern distribution	Deff (effective diffusion), CSF bulk flow, kel, CNS, ISF–CSF exchange, tortuosity factor	Brain parenchyma/CSF → local diffusion/convection; minimal systemic exposure	Spatial or multi-CSF compartment model preferred; clearance via arachnoid villi/perivascular pathways
Transdermal	[Absorption sub-model] Multi-layer skin compartments (stratum corneum, viable epidermis, dermis); sustained lag-phase input to systemic circulation	Pskin, lag time (tlag), patch surface area, vehicle–skin partition coefficient, kdepot	Skin depot → dermis capillaries → systemic → BBB	Slow sustained input smooths plasma peaks; stratum corneum permeation depends on lipophilicity

**Table 4:** Summary of DNC classes and example chemotherapeutics investigated for brain delivery.

DNC Category	Advantages	Representative Therapeutic Agents
**Polymersome** [54,55,56,57,58,59,60]	Controlled and sustained drug release. Versatile for encapsulating various drugs. Functionalized for active targeting. Can cross the BBB via receptor-mediated transport.	3-bis(2-chloroethyl)-1-nitrosourea, doxorubicin, methotrexate, temozolomide, gemcitabine, paclitaxel (oncology); curcumin, rivastigmine (Alzheimer’s disease); dopamine (Parkinson’s disease)
**Liposome** [61,62,63]	Biocompatible and biodegradable, with the ability to encapsulate a wide range of drug molecules, and be functionalized for targeted delivery to brain cells	Doxorubicin, methotrexate, cisplatin, irinotecan, topotecan, paclitaxel (oncology); donepezil (Alzheimer’s disease); valproic acid (epilepsy)
**Iron oxide nanoparticles** [64,65]	Superparamagnetic properties enabling magnetic guidance and MRI contrast; high drug-loading capacity; surface modifiable for brain targeting; biocompatible with proper coatings.	Doxorubicin, cisplatin, paclitaxel (oncology); levodopa (Parkinson’s disease)
**Gold nanoparticles** [24,66,67]	High surface-area-to-volume ratio; easy conjugation of targeting ligands and drugs; good photothermal properties for combined therapy; can cross the BBB with appropriate coatings.	Doxorubicin, methotrexate, cisplatin, paclitaxel (oncology); β-amyloid siRNA (Alzheimer’s disease)
**Quantum dots** [68,69,70]	Photoluminescent, photostable, tunable emission/excitation spectra, visualization of individual molecules, readily monitor drug delivery, and low toxicity.	Topotecan, doxorubicin, temozolomide (oncology); nerve growth factor (neurodegeneration)
**Dendrimer** [64,71,72]	High molecular uniformity, monodispersity, kinetic stability, abundant free functional groups, and low toxicity.	Methotrexate, doxorubicin, temozolomide, docetaxel, arsenic trioxide (oncology); BDNF (neuroprotection); valproic acid (epilepsy)
**Nanogel** [65]	Serum stability, uniformity, fluid-like transport of nanogel properties, bioadhesive, biocompatible, biodegradable, deformable, stimulus-responsive release, and low toxicity.	Doxorubicin, cisplatin, methotrexate, paclitaxel (oncology); memantine (Alzheimer’s disease)

**Table 5:** PBPK parameter classification and examples.

Parameter Category	Name	Description
**Anatomical parameters**	Body weight and organ weights	The total body weight and the weights of individual organs
	Tissue volumes	Volumes of different tissues and organs (e.g., liver, kidney, heart)
	Blood flow rates	The rate at which blood flows through various organs and tissues.
**Physiological parameters**	Cardiac output	The volume of blood the heart pumps per minute
	Tissue perfusion rates	The rates of blood flow through specific tissues
	Ventilation rate	The rate of air intake into the lungs
**Biochemical parameters**	Enzyme activity levels	Levels of enzymes involved in metabolism
	Plasma protein binding	The extent to which a substance binds to proteins in the blood
	Partition coefficients	Ratios that describe how a substance distributes between different tissues and the blood
**Chemical-specific parameters**	Absorption rates	The rate at which a substance is absorbed into the bloodstream from the site of administration
	Metabolic rates	The rate at which a substance is metabolized or broken down in the body
	Excretion rates	The rate at which a substance is excreted from the body
**Transport parameters**	Permeability coefficients	Rates at which a substance crosses cellular membranes
	Transporter activity levels	Activity levels of specific transporters that facilitate the movement of substances into and out of cells
**Application-specific parameters**	Dosing regimen	The amount, frequency, and duration of drug administration
	Route of administration	The path by which a drug is taken into the body

**Table 6:** Methods of obtaining PBPK parameters.

Methodology	Identification	Description
**Experimental Methods**	In Vivo Studies	Animal Studies: Conducting pharmacokinetic studies in animals to obtain anatomical, physiological, and biochemical parameters. Human Studies: Clinical trials in humans to determine parameters like absorption rates, metabolic rates, and excretion rates.
	In Vitro Studies	Cell Culture: Using human or animal cells to study metabolism, transport, and binding. Enzyme Kinetics: Measuring enzyme activity levels using liver microsomes or recombinant enzymes. Protein Binding Assays: Determining the extent of plasma protein binding using techniques like equilibrium dialysis or ultrafiltration.
**Computational Methods**	In Silico Predictions	QSAR Models (Quantitative Structure-Activity Relationship): Using chemical structure to predict pharmacokinetic parameters. Physiological Databases: Using databases like Simcyp or PK-Sim to obtain physiological and anatomical parameters.
	Mechanistic Modeling	Using previously developed models to estimate parameters for new substances. Scaling parameters from animals to humans using allometric scaling.
**Literature-Based Methods**	Reviewing Published Data	Extracting relevant parameters from the scientific literature, including previous studies and reviews. Using meta-analyses and systematic reviews to compile data from multiple sources.
	Databases	Accessing pharmacokinetic databases (e.g., DrugBank, PubMed) to obtain previously reported parameters. Utilizing population databases for demographic-specific parameters.
**Estimation and Fitting Methods**	Parameter Estimation	Using optimization techniques to fit model parameters to experimental data. Applying statistical methods like nonlinear regression or Bayesian approaches.
	Sensitivity Analysis	Identifying the most influential parameters through sensitivity analysis to prioritize experimental efforts.

**Table 7:** Examples of PK databases.

Database Name	Data Type	ML Potential Application in Data Collection
**Curated diverse molecular database** [102]	Log BB	ML can screen a wide array of drug candidates computationally to predict their brain distribution profiles, allowing researchers to focus only on the most promising candidates for further testing.
**Brainpeps** [103]	Log BB	
**PK/DB** [104]	Log BB	
**ChEMBL** [105]	Log BB	
**Pub-Chem** [106]	Log BB	
**Obach et al.’s dataset** [107]	PK parameters	ML models can integrate and analyze existing data from various sources (e.g., previous studies, public databases) to generate new insights, reducing the need for additional data collection.
**Konovalov’s dataset** [108]	PK parameters	
**Database for partition of volatile organic compounds** [109]	Binary classification (BBB+/BBB−)	ML models can integrate and analyze existing data from various sources (e.g., previous studies, public databases) to generate new insights, reducing the need for additional data collection.
**Therapeutics Data Commons (TDC)** [110]	Open ML-ready benchmark platform; 66 curated datasets across 22 drug-discovery tasks, including ADMET and BBB endpoints	ML models can integrate and analyze existing data from various sources (e.g., previous studies, public databases) to generate new insights, reducing the need for additional data collection.
**BBB benchmark (BBB_Martins)** [111]	Binary classification (BBB+/BBB−); 1975 compounds with predefined scaffold train/test splits	
**PK-DB** [112]	AUC of brain/plasma	

**Table 8:** Conventional PBPK research summary.

Predicted Property	Key PBPK Model Features	Performance	Limitations and ML’s Potential
**Blood–brain barrier permeability** [23]	Multi-compartment PBPK model; brain partitioned into vascular and tissue sub-compartments; passive transcellular diffusion + P-glycoprotein-mediated active efflux; key inputs: plasma protein binding, tissue partition coefficients (Kp), cerebral blood flow	R^2^ = 0.941; blood–brain barrier permeability	The brain’s microenvironment is highly heterogeneous, with varying tissue types, BBB permeability, and interstitial fluid dynamics. Accurately modeling drug diffusion across these diverse regions is challenging. ML models excel at capturing complex, nonlinear relationships that are difficult to model with traditional PBPK approaches. This makes them particularly suited for modeling the nonlinear kinetics of drug diffusion in the brain.
**Blood–brain barrier permeability** [121]	Two-compartment brain PBPK model; passive transcellular diffusion across BBB; key inputs: lipophilicity (log *p*), molecular weight, H-bond donors/acceptors, plasma unbound fraction	R^2^ = 0.62; blood–brain barrier permeability	
**Blood–brain barrier permeability** [122]	Physiologically based flow-limited distribution model; brain homogenate partition coefficient; key inputs: Kp, brain, unbound fraction in plasma (fu, *p*), blood clearance	AUC 0.92; blood–brain barrier permeability	
**Blood–brain barrier permeability** [93]	PBPK with permeability-surface area (PS) product; accounts for active BBB efflux transport; key inputs: PS_BBB, fu, brain, Kp, uu, brain, cerebral blood flow	AUC 0.71; blood–brain barrier permeability	
**Blood–brain barrier permeability** [123]	Multi-compartment PBPK: brain vascular, ISF, and intracellular sub-compartments; P-gp efflux incorporated; key inputs: Kp, uu, brain, Kp, uu, CSF, active efflux clearance (CLefflux)	AUC 0.83; blood–brain barrier permeability	
**Blood–brain barrier permeability** [108]	Mechanistic brain distribution model; passive diffusion and active transport mechanisms; key inputs: fu, brain, active transport clearance (PSactive), BBB permeability coefficient	Blood–brain barrier permeability	
**Blood–brain barrier permeability** [124]	In vitro-calibrated PBPK model; integrates MDCK/Caco-2 assay permeability data; key inputs: Papp (in vitro BBB permeability), protein binding, intrinsic clearance (CLint)	RMSE 0.258; blood–brain barrier permeability	

**Table 10:** ML methods and their properties.

Method	Application	Advantage	Disadvantage
**Linear Regression**	Predictions.	Transparency in providing relevant and significant information about physicochemical descriptors.	Limited to linear relationships. Easily affected by outliers [22].
**RF**	Classifying drug molecules and nanoparticles based on their physicochemical properties, such as size, shape, surface charge, and functionalization.	Automatic selection of input variables; removal of insignificant descriptors; extraction of informative features from small, noisy, and heterogeneous datasets.	Cannot handle non-numerical inputs directly; training time is relatively long compared with simpler models; prone to overfitting when the number of trees or tree depth is not tuned.
**SVM**	Classifying and predicting the targeting efficiency of nanoparticles to specific tissues or cells.	High precision in high-dimensional feature spaces; supports both classification and regression tasks. Handles nonlinear relationships, collinear descriptors, and small datasets while mitigating overfitting through regularization.	The performance of SVM heavily depends on the choice of the kernel function and its parameters, which require careful tuning and cross-validation. SVM can be sensitive to noisy data and outliers, which can affect the placement of the optimal hyperplane and degrade performance.
**GBM**	Identifying the most relevant features influencing the behavior and efficacy of nanomaterials.	It iteratively boosts weak trees to capture subtle nonlinear interactions while still being relatively interpretable and sample-efficient [147].	Despite regularization, GBM can still overfit, especially if the model is too complex or the number of boosting rounds is too high.
**ANN**	Capturing intricate, nonlinear relationships within data, making them suitable for complex biological processes in nanomedicine.	Handles the nonlinear nature of structure–activity relationships and large descriptor datasets, including unnecessary variables.	Prone to overfitting, especially with small datasets, leading to models that perform well on training data but poorly on unseen data. Has numerous hyperparameters that need to be carefully tuned, which can be a complex and time-consuming process.
**Bayesian**	Estimating the physicochemical properties of nanoparticles and their uncertainties.	Allows for the incorporation of prior information, which can be particularly valuable in nanomedicine, where prior biological knowledge and previous experimental results are available. Robustness to overfitting.	Markov Chain Monte Carlo (MCMC) methods, commonly used for Bayesian inference, can suffer from convergence issues, especially for complex models.

**Table 11:** Software tools for ADME modeling.

Name	Description	Limitation
**PBPK Plus (part of GastroPlus X.2, Simulations Plus)** [153]	Commercially available software that simulates the distribution and elimination of a compound throughout the body and tracks concentrations in any tissue.	It could be limited in its ability to handle detailed mechanistic models. May not cover all therapeutic areas (e.g., oncology, rare diseases).
**BIOVIA Discovery Studio 2026 (Dassault Systèmes)** [154]	Commercially available software used for predicting ADMET properties of a drug.	Limited in PBPK-focused modeling; more aligned with molecular modeling, so PK applications might be secondary. Requires extensive user expertise for effective use in PK studies.
**Simcyp Simulator V25 (Certara)** [155]	PBPK software used for determining first-in-human dosing, optimizing clinical study design, evaluating new drug formulations, setting the dose in untested populations, performing virtual bioequivalence analyses, and predicting drug–drug interactions (DDIs).	High cost can be prohibitive for smaller institutions or individual researchers. A complex interface may require extensive training.
**The BIOSIM 0.0.12** [156]	An ML biosimulation software platform, which includes a semimechanistic model of in vivo physiology with 14 individual compartments corresponding to important organs in the body.	Limited visibility and adoption compared to more established platforms. Potential lack of integration with widely used pharmacokinetic data repositories.
**ACD/Labs Percepta Platform, Version 2025** [157]	Platform for predicting aqueous solubility, boiling point, logD, logP, pKa, Sigma, and other molecular descriptors for organic compounds, from structure.	Primarily focused on physicochemical properties; it may not support full PBPK modeling workflows. Potentially lacks advanced features for simulating physiological conditions.
**ADMET Predictor 13 (Simulations Plus)** [158]	Software that provides strategic pharmacology and pharmacometric consulting services, and PK/PD model development with NONMEM.	Primarily focused on ADMET properties, limiting its use in detailed physiological PK simulations. Limited interactivity and customization options for complex scenarios.
**PK-Sim v12 (Open Systems Pharmacology)** [159]	Powerful and easy-to-use modeling and simulation tools for pharmaceutical and other life-sciences applications.	May lack extensive support for all organ systems and drug distribution mechanisms. Limited in population variability modeling (e.g., different ethnicities, disease states).

## Data Availability

No new data were created or analyzed in this study. Data sharing is not applicable to this article.

## Abbreviations

PBPK	physiologically based pharmacokinetics
ML	machine learning
DNC	drug-loaded nanocarrier
BBB	blood–brain barrier
ODE	ordinary differential equation
ADME	absorption, distribution, metabolism, and excretion
QSAR	quantitative structure–activity relationship
PRISMA	Preferred Reporting Items for Systematic Reviews and Meta-Analyses
MRI	magnetic resonance imaging
BCSFB	blood–cerebrospinal fluid barrier
LMEM	linear mixed-effect model
SVM	support vector machine
RF	random forest
RNN	recurrent neural network
ISF	interstitial fluid
LightGBM	gradient boosting machine learning algorithm
NHD	number of hydrogen bonding donor
NHA	number of hydrogen bonding acceptor
RFE	recursive feature elimination
ACC	accuracy
MD	molecular descriptor
EXT	extra-tree classifier
XGB	XGBoost
